# Integrating Bioinformatics Tools to Handle Glycosylation

**DOI:** 10.1371/journal.pcbi.1002285

**Published:** 2011-12-29

**Authors:** Yuliet Mazola, Glay Chinea, Alexis Musacchio

**Affiliations:** Department of Bioinformatics, Center for Genetic Engineering and Biotechnology, Havana, Cuba; Whitehead Institute, United States of America

This is an original *PLoS Computational Biology* tutorial.

## Introduction

This tutorial is planned for biologists and computational biologists interested in bioinformatics applications to study protein glycosylation. Glycosylation is a co- and post-translational modification that involves the selective attachment of carbohydrates to proteins. The enhancement of glycosylation by applying glycoengineering strategies has become widely used to improve properties for protein therapeutics. In this tutorial, the use of bioinformatics to assist the rational design and insertion of N-glycosylation sites in proteins is described.

## Background

**Figure 1:**
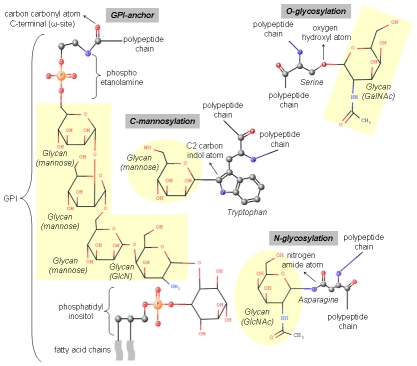
Schematic representation of glycosylation forms. For each glycosylation type, the amino acid acceptor site is illustrated in balls and sticks: *N*-glycosylation (asparagine residue), *O*-glycosylation (serine residue), *C*-mannosylation (tryptophan residue), and glycosylphosphatidylinositol (GPI) anchor (C-terminal protein residue). Small balls colored in grey, red, blue, and orange represent carbon, oxygen, nitrogen, and phosphorus atoms, respectively. Hydrogen atoms were not shown. The atoms involved in glycan linkage are indicated with rows. Glycan molecules are shown as sticks and highlighted with a yellow background color. The GPI molecule was divided into three parts: phosphoethanolamine, glycan core, and phosphatidylinositol. The glycan core is composed of one non-acetylated glucosamine (GlcN) and three mannose moieties. The long fatty acids contained in the phosphatidylinositol portion are indicated using waves.

Glycosylation is a co- and post-translational modification involving the covalent addition of carbohydrates to proteins. Carbohydrates (also referred to as glycans, sugars, or saccharides) are adopting linear and branched structures and are composed of monosaccharides, which are covalently linked by glycosidic bonds. There are four enzymatic glycosylation processes: N-glycosylation, *O*-glycosylation, *C*-glycosylation (or *C*-mannosylation), and glycosylphosphatidylinositol (GPI) anchor (Figure 1). Glycan acceptor sites for each glycosylation type are described in Table 1. Experimental detection of occupied glycosylation sites in proteins is an expensive and laborious process [1]. Instead, a number of glycosylation prediction methods as well as glycan and glycoprotein analysis tools have been developed (Table 2 and Table 3). For a detailed description of glycobiology-related databases and software, including glycosylation predictors, the reader is referred to nice reviews on the subject [2]–[5].

**Table 1:** General features of different glycosylation types.

Glycosylation Type	Glycosylation Sequences Motifs	Glycosylation Acceptor Site	Organism	Reference
*N*-glycosylation	In eukaryotes, glycan molecules are attached to the asparagine residue from sequons: Asn-x-Ser and Asn-x-Thr, or in some rare cases in Asn-x-Cys where x is not a proline residue. In prokaryotes, the sequon is extended to Asp/Glu-z-Asn-x-Ser and Asp/Glu-z-Asn-x-Thr, where x and z are not proline residues.	Nitrogen atom from the amide group in the asparagine residue	Eukaryotes and prokaryotes	[30], [31]
*O*-glycosylation	No specific sequence motifs have been defined. Sugars are attached to serine and threonine residues usually found in a beta conformation and in close vicinity to proline residues.	Oxygen atom from the hydroxyl group in serine or threonine residues	Eukaryotes and prokaryotes	[32]–[34]
*C*-glycosylation	Carbohydrates are attached to the first tryptophan residue from the following motifs: Trp-x-x-Trp, Trp-x-x-Phe, Trp-x-x-Tyr, and Trp-x-x-Cys. Any amino acid could be placed at the x position, although small and/or polar residues are preferred, such as alanine, glycine, serine, and threonine.	Carbon atom (C2) from the indole group in the tryptophan residue	Eukaryotes except yeast	[35]–[39]
GPI anchor	A specific C-terminal signal sequence is recognized and cleaved, creating a new C-terminal protein end (ω-site). The GPI molecule is added to the ω-site. No consensus sequence for ω-site localization has been described. Typical residues in ω-site include: cysteine, aspartic acid, glycine, asparagine, and serine.	Carbon atom from the C-terminal carbonyl group at the ω-site	Eukaryotes and a reduced subset of archaea	[40]–[42]

GPI, glycosylphosphatidylinositol.

**Table 2:** Glycosylation prediction servers.

Server	Portable Version	Method	Description	URL
NetNGlyc	Only for academics	ANN	*N*-glycosylation	http://www.cbs.dtu.dk/services/NetNGlyc/
EnsembleGly	No	SVM	*N*-glycosylation	http://turing.cs.iastate.edu/EnsembleGly/
EnsembleGly	No	SVM	*O*-glycosylation	http://turing.cs.iastate.edu/EnsembleGly/
EnsembleGly	No	SVM	*C*-glycosylation	http://turing.cs.iastate.edu/EnsembleGly/
GPP	No	RFM	*N*-glycosylation	http://comp.chem.nottingham.ac.uk/glyco/
GPP	No	RFM	*O*-glycosylation	http://comp.chem.nottingham.ac.uk/glyco/
NetOGlyc	Only for academics	ANN	*O*-glycosylation	http://www.cbs.dtu.dk/services/NetOGlyc/
Oglyc	No	SVM	*O*-glycosylation	http://www.biosino.org/Oglyc/
CKSAAP_OGlySite	No	SVM	*O*-glycosylation	http://bioinformatics.cau.edu.cn/zzd_lab/CKSAAP_OGlySite/
YinOYang	Only for academics	ANN	*O*-glycosylation	http://www.cbs.dtu.dk/services/YinOYang/
Big-PI	No		GPI anchor	http://mendel.imp.ac.at/gpi/gpi_server.html
GPI-SOM	Yes	ANN	GPI anchor	http://gpi.unibe.ch/
FragAnchor	No	ANN and HMM	GPI anchor	http://navet.ics.hawaii.edu/~fraganchor/NNHMM/NNHMM.html
PredGPI	Only for academics upon request	HMM and SVM	GPI anchor	http://gpcr.biocomp.unibo.it/predgpi/
NetCGlyc	Only for academics	ANN	*C*-mannosylation	http://www.cbs.dtu.dk/services/NetCGlyc/

ANN, artificial neural network; SVM, support vector machine; RFM, random forest method; HMM, hidden Markov model.

**Table 3:** Tools for glycan and glycoprotein analysis.

Tools	Portable Version	Description	URL
GlyProt	No	Modeling 3D structure of glycoproteins with attached *N*-glycans	http://www.glycosciences.de/modeling/glyprot/php/main.php
SWEET-II	No	Building 3D carbohydrate models	http://www.glycosciences.de/modeling/sweet2/
Glydict	No	Prediction of *N*-glycan 3D structures	http://www.glycosciences.de/modeling/glydict/
Shape	Yes	Prediction of carbohydrate conformational space	http://sourceforge.net/projects/shapega/
GlySeq	No	Statistical analysis of residues neighboring *N*-glycan sequons in protein sequence	http://www.glycosciences.de/tools/glyseq/
GlyVicinity	No	Statistical analysis of residues surrounding carbohydrate chains in protein 3D structure	http://www.glycosciences.de/tools/glyvicinity/

3D, three-dimensional.

## The Attractiveness of Modifying Protein Glycosylation

**Figure 2:**
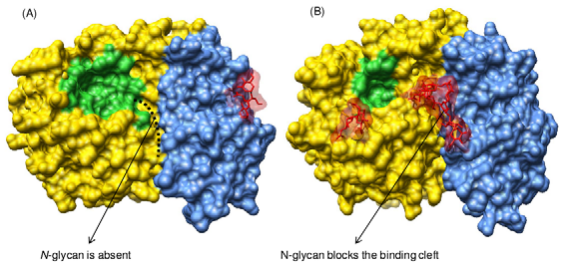
Three-dimensional structures of two glycosyl hydrolase 32 (GH32) family enzymes. Surface representation of the overall 3D structure of (A) *Arabidopsis thaliana* cell-wall invertase (PDB database accession code: 2AC1) and (B) *Cichorium intybus* fructan 1-exohydrolase IIa (PDB database accession code: 1ST8). The N- and C-terminal domains are colored in yellow and blue, respectively. The attached *N*-glycan molecules are represented as sticks in red color. The active site is shown in green. Another binding pocket that extends between N- and C-terminal domains is orange, highlighted in (A). This cleft is reserved for higher DP-inulin type fructans. An open conformation of the mentioned cavity is observed in GH32 enzymes capable of degrading inulin substrates, such as *C. intybus* fructan 1-exohydrolase IIa (A). However, the introduction of a glycosyl chain blocks the cleft and prevents inulin binding and degradation in some GH32 enzymes, such as in *A. thaliana* invertase (B).

Of particular interest is the role of carbohydrates in modulating physico-chemical and biological properties of proteins. Several glycosylation parameters are involved, including the number of glycans attached, the position of the glycosylation sites, and the glycan features (such as the molecular size, sequence, and charge). Glycan can influence protein function [6]; the presence of a glycosyl chain pointing toward a binding pocket might block such a cavity and hence, influence the ligand binding mode and affect protein biological activity (Figure 2). Carbohydrates can also increase protein stability and solubility, as well as reduce immunogenicity and susceptibility to proteolysis [7]. This explains why the rational manipulation of glycosylation parameters (glycoengineering) is widely applied to obtain proteins suited for therapeutic applications [8]. Glycoengineering can enhance in vivo activity even in proteins that do not normally contain N-glycosylation sites [9]. Some protein instabilities prevented by applying glycosylation engineering include proteolytic degradation, formation of crosslinked species, unfolding processes, oxidation, low solubility, aggregation, and kinetic inactivation [10].

## Rational Design and Insertion of N-glycan Sites in Proteins

**Figure 3:**
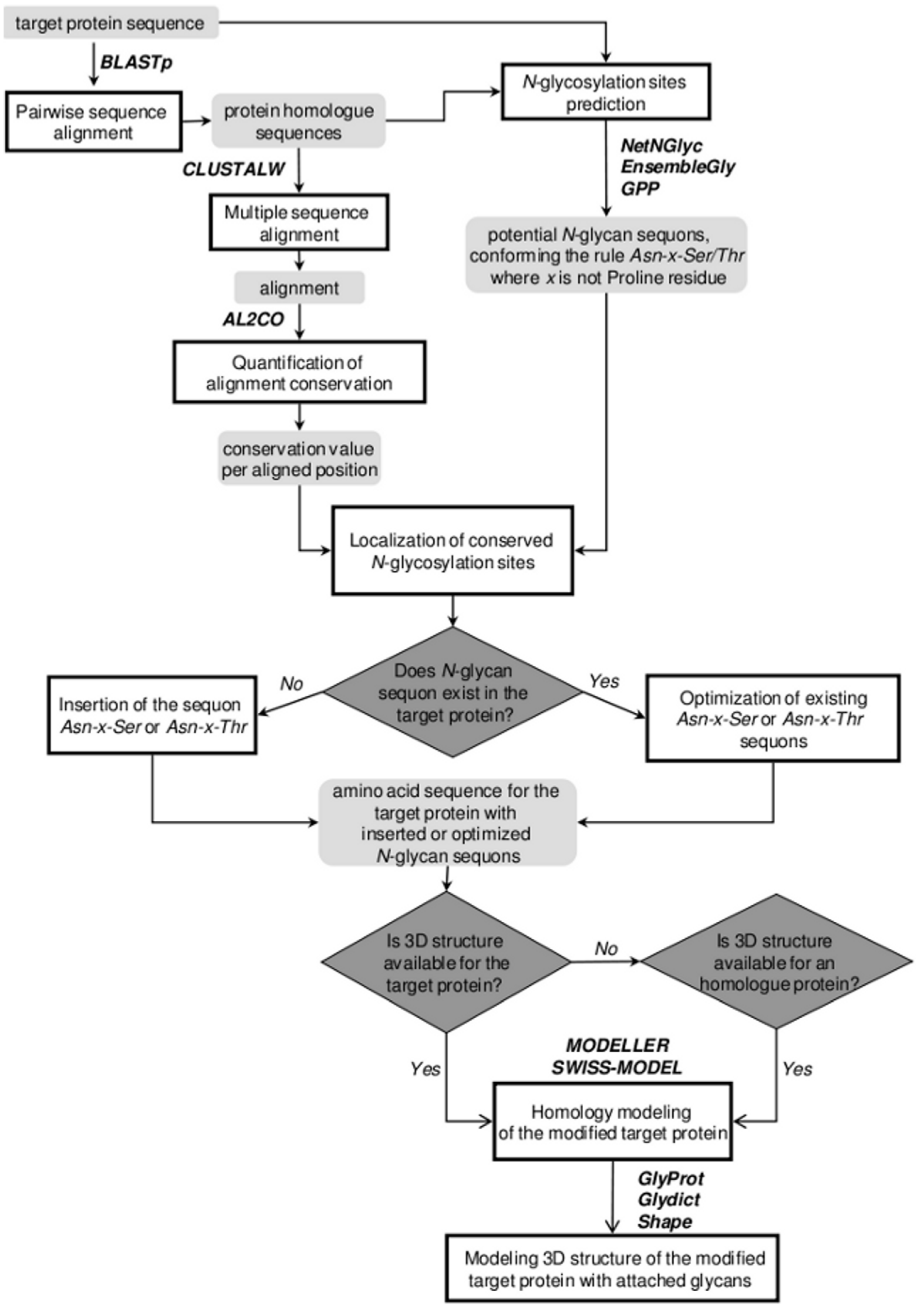
Workflow for rational design and insertion of *N*-glycan sites in proteins.

One of the strategies used in glycoengineering involves the introduction of N-glycosylation sequons to increase carbohydrate content in protein pharmaceuticals [7]. In this tutorial, a workflow for the rational design and insertion of N-glycan sites into a desirable protein (also referred to as a target protein) using bioinformatics is provided (Figure 3). A detailed description of the workflow is given below. General features and availability of non-glycobiology-related bioinformatics resources can be found in Table 4.

**Table 4:** Software for protein sequence and tertiary structure analysis.

Software	Portable Version	Description	URL
BLASTp	Yes	Pairwise sequence alignment comparing a query protein sequence with a database of protein sequences	http://blast.ncbi.nlm.nih.gov/Blast.cgi
CLUSTALW	Yes	Multiple sequence alignment comparing a number of protein sequences	http://www.ebi.ac.uk/Tools/msa/clustalw2/
AL2CO	Yes	Quantification of conservation index at each aligned position in a multiple sequence alignment	http://prodata.swmed.edu/al2co/al2co.php
VMD	Yes	Molecular visualization for displaying, animating, and analyzing large biomolecular systems using 3D graphics	http://www.ks.uiuc.edu/Research/vmd/
PSI-PRED	Yes	Prediction secondary structure from amino acid sequence	http://bioinf.cs.ucl.ac.uk/psipred/
MODELLER	Free for academics, but commercial versions are also available.	Homology modeling of protein 3D structures. An option to introduce single point mutations in the target protein to obtain its 3D model is also included.	http://www.salilab.org/modeller/
SWISS-MODEL	No	Automated comparative modeling of 3D protein structures	http://swissmodel.expasy.org/
GROMACS	Yes	Molecular dynamics simulations	http://www.gromacs.org/

3D, three-dimensional.

The target protein amino acid sequence is the starting point in this analysis. Additional information, such as post-translational modifications, site-directed mutagenesis studies, and three-dimensional (3D) structure, are also helpful. These data can be found in the protein annotation and literature databases UniProtKB [11] and PubMed [12], respectively.

Prior to performing any modification to the target protein sequence, one should know the residues involved in protein function and tertiary structure. These residues should not be modified. In general, functional and structural relevant residues tend to be more conserved within a protein family [13]. Conserved residues are identified by multiple sequence alignment using, for example, the CLUSTALW server [14], analyzing the sequence similarity among the target protein and its homologues. In particular, a multiple sequence alignment with diverse and divergent protein homologue sequences is suggested, since residues conserved over a longer period of time are under stronger evolutionary constraints. The homologue proteins are recognized via a pairwise alignment using, for instance, the BLASTp server [15]. A degree of conservation for each aligned position in the multiple sequence alignment is quantified. At this step, available tools for sequence conservation analysis could be applied, like the AL2CO server [16]. The amino acid frequencies for each aligned position are estimated and the conservation index is calculated from those frequencies. As input for the AL2CO server, the multiple sequence alignment file is required. Optionally, if a Protein Data Bank (PDB) file (atomic coordinates) of the target or any related homologue protein is also uploaded, the AL2CO server adds the calculated conservation indices into the output PDB file. Then, conserved motifs can be mapped onto the 3D structure and visualized with the Visual Molecular Dynamics (VMD) software [17].

We recommend the insertion of N-glycan sites, such as Asn-x-Ser/Thr, preferentially at positions where potential N-glycosylation sequons predominate in the homologue proteins. The prediction of N-glycosylation sites has to be done for the target and homologue proteins, and any of the available prediction servers, such as NetNGlyc, EnsembleGly, or GPP, can be used (Table 2). The GPP server input is the protein amino acid sequence and the output is sent by email. For NetNGlyc and EnsembleGly servers, the protein UniProtKB/Swiss-Prot accession number or primary amino acid sequences are accepted as input. Results are shown online and are easy to understand. Predicted N-glycan sites are mapped and scored onto the protein sequence representing the occurrence probability of N-glycosylation. In the case of NetNGlyc, the predicted Asn-x-Ser/Thr motifs are highlighted in red color, and a graph showing potential N-glycosylation versus amino acids position is also given.

Following the glycosylation prediction, three potential cases may emerge: (a) predicted N-glycan sites are found in both the target and the homologue proteins; (b) predicted N-glycan sites are found only in homologue proteins; and (c) no N-glycan sites are predicted either in the target protein or in homologue proteins. How to proceed?

**Figure 4:**
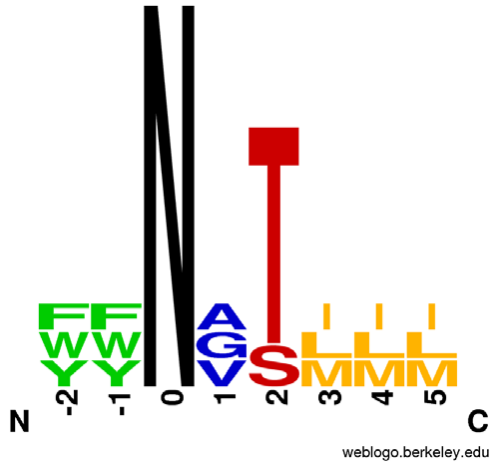
Amino acid preferences in occupied *N*-glycan sites. The sequence logo displays residues preferentially placed at occupied *N*-glycan sequons. Neighboring residues located downstream (positions +3 to +5) and upstream (positions −1 and −2) from the asparagine residue (position 0) are also shown. The size of each letter represents the residue prevalence at the putative position. For example, threonine residue is preferred over serine, at position +2. The WebLogo server [29] was used to generate the sequence logo.

In case (a), an optimization of Asn-x-Ser/Thr sequons replacing residues at position +1 (Asn occupies position 0) or surrounding the sequon is done. Statistical analysis of occupied and non-occupied N-glycosylation sites revealed that the amino acids at position +1 and nearby N-glycan sequons modulate the occurrence of N-glycosylation (Table 5). Some suggestions for amino acid substitutions: (a) aromatic amino acids (phenylalanine, tyrosine, and tryptophan) in position −2 and −1, (b) small nonpolar amino acids (glycine, alanine, and valine) in position +1, and (c) bulky hydrophobic amino acids (leucine, isoleucine, and methionine) in positions +3 to +5 (Figure 4). The statistical analysis of amino acids neighboring N-glycosylation sites in the protein primary sequence and tertiary structure can be conducted using the GlySeq and GlyVicinity software, respectively [18].

**Table 5:** Comparative studies for occupied and non-occupied *N*-glycan sites.

Description	Reference
Influence of proline residue neighboring the Asn-x-Ser and the Asn-x-Thr sequons over *N*-glycosylation in the yeast invertase protein.	[43]
Relevance of certain amino acid substitutions at the position +1 in the Asn-x-Ser sequon for *N*-glycosylation efficiency in the rabies virus glycoprotein.	[44]
Relevance of certain amino acid substitutions at the position +1 in the Asn-x-Ser and the Asn-x-Thr sequons for *N*-glycosylation efficiency using different variants of rabies virus glycoprotein.	[45]
Influence of the 20 amino acids at the position following the Asn-x-Ser and Asn-x-Thr sequons for *N*-glycosylation efficiency using different variants of rabies virus glycoprotein.	[46]
Occurrence frequency analysis of some amino acid residues at position +1 in the Asn-x-Ser and Asn-x-Thr sequons studying glycoproteins from the PDB database [47].	[48]
Influence of the 20 amino acids flanking the upstream and downstream of Asn-x-Ser and Asn-x-Thr sequons, using glycoproteins from the UniProtKB/Swiss-Prot database [11].	[19]
Primary, secondary, and tertiary structures statistical analysis of occupied and non-occupied *N*-glycosylation sites using glycoproteins from the PDB database [47].	[49]

In case (b), a sequence pattern like Asn-x-Ser or Asn-x-Thr is inserted in the target protein. There is a large preference for threonine, as opposed to serine, in position +2. This is in agreement with the observation that replacing serine with threonine in the sequon results in an overall increase of the occupancy [19]. Some suggestions for amino acid substitution at position +1 are (a) highly conserved amino acids at the position +1 within the homologue proteins may be kept except proline, and (b) small nonpolar amino acids (glycine, alanine, and valine) at the position +1 increase the probability of sequon occupancy [20].

In case (c), the analysis of the secondary structure has to be performed to insert the N-glycan sites at or just after protein secondary structure changes. Glycosylation sites are frequently found in points of changes of secondary structure, with a bias toward turns and bends [19]. Protein secondary structure features are described in the PDB file. If no 3D structures are available, a prediction of the secondary structure can be solved using, for example, the PSI-PRED server [21]. Only the primary amino acid sequence is required as input.

With the insertion of N-glycosylation sites in the target protein primary structure, the attachment of N-glycan molecules is favored. Then, the analysis and visualization of the glycoprotein is also helpful. Tertiary glycoprotein structure having attached N-glycans can be modeled using the GlyProt server [22]. This facilitates the identification of spatially unfavorable N-glycosylation sites [6].

The 3D glycan structures are provided in the GlyProt server database; they can also be implemented using the SWEET-II [23], Glydict [24], and Shape [25] software. For the GlyProt server input 3D protein structure, the atomic coordinate file from the modified target protein is required. In this case, a 3D structure model has to be built, using the structure of the native target protein or related homologue as a template. The sequence used as input to build the 3D model has to contain the inserted N-glycan sequons, for which homology modeling software like MODELLER [26] and the online SWISS-MODEL server [27] can be used.

Finally, molecular dynamics simulations to explore protein backbone conformational changes could be applied using, for example, the GROMACS software [28]. This strategy allows for the refinement of the initial glycoprotein structure. All bioinformatics software previously mentioned are freely available. An example of the application of the workflow presented in this manuscript is available in Supporting Information (Text S1 and Figures S1, S2, S3, S4).

## Concluding Remarks

In a brief survey, a workflow integrating available bioinformatics resources to assist protein glycosylation was exposed. In particular, the rational manipulation of the native N-glycosylation pattern, including in silico tools, was given. The application of the bioinformatics strategy described in this tutorial, at the early stages of glycoengineering, can help the design and insertion of N-glycan sites in proteins, reducing time, effort, and cost.

## Supporting Information

Figure S1 Protein tertiary structure.
(TIF)

Figure S2 Multiple sequence alignment.
(PDF)

Figure S3 Pairwise sequence alignment.
(PDF)

Figure S4 Protein tertiary structure with modeled N-glycans.
(TIF)

Text S1 Supporting information text.
(DOC)
