# Supplementary figures and images for: Integrating Bioinformatics Tools to Handle Glycosylation
Source: PLoS Comput Biol. 2011 Dec 29;7(12):e1002285. doi: 10.1371/journal.pcbi.1002285 (PMC3248387; doi:10.1371/journal.pcbi.1002285)

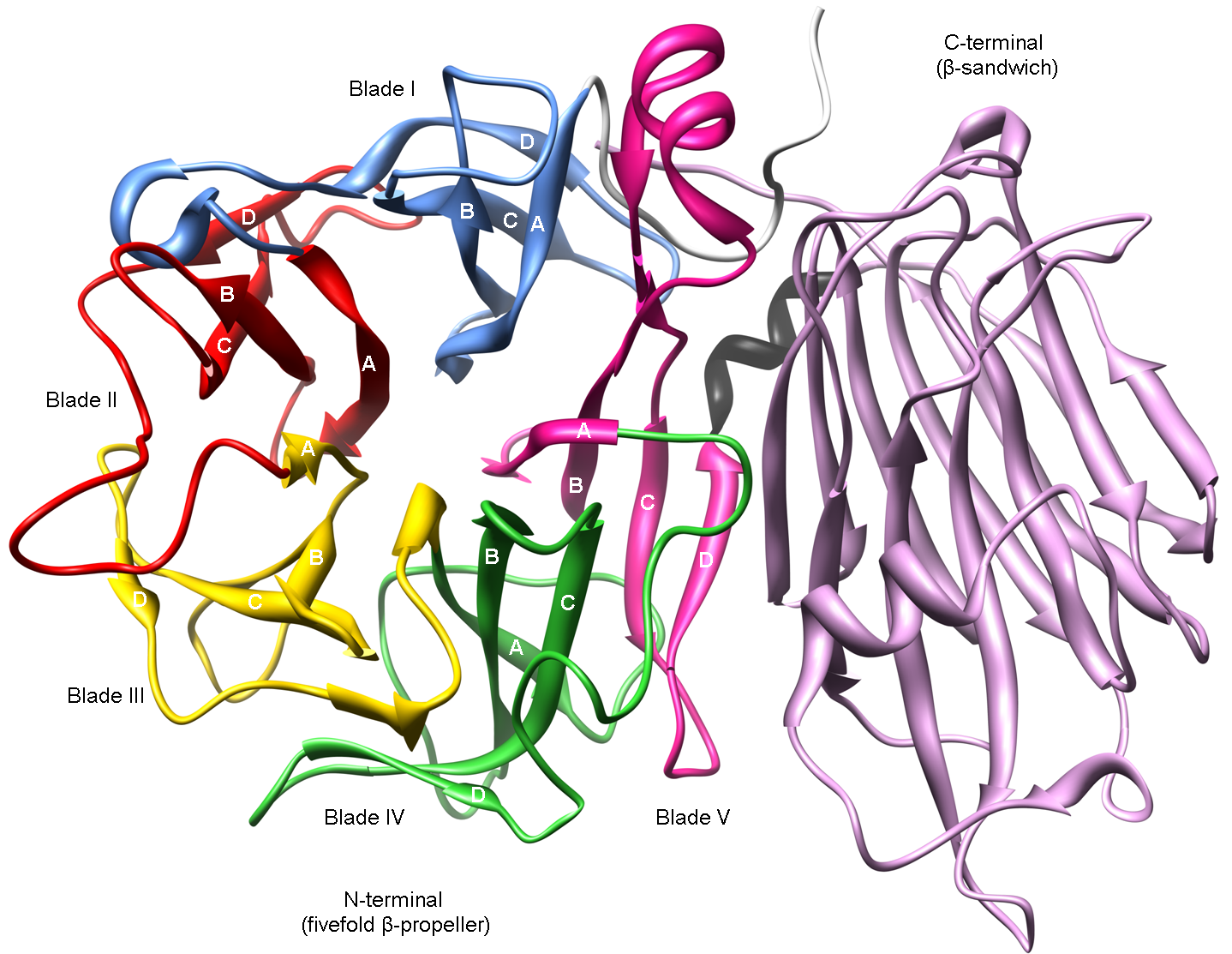

Supplement: Figure S1 — Protein tertiary structure. (TIF) [file pcbi.1002285.s001.tif]

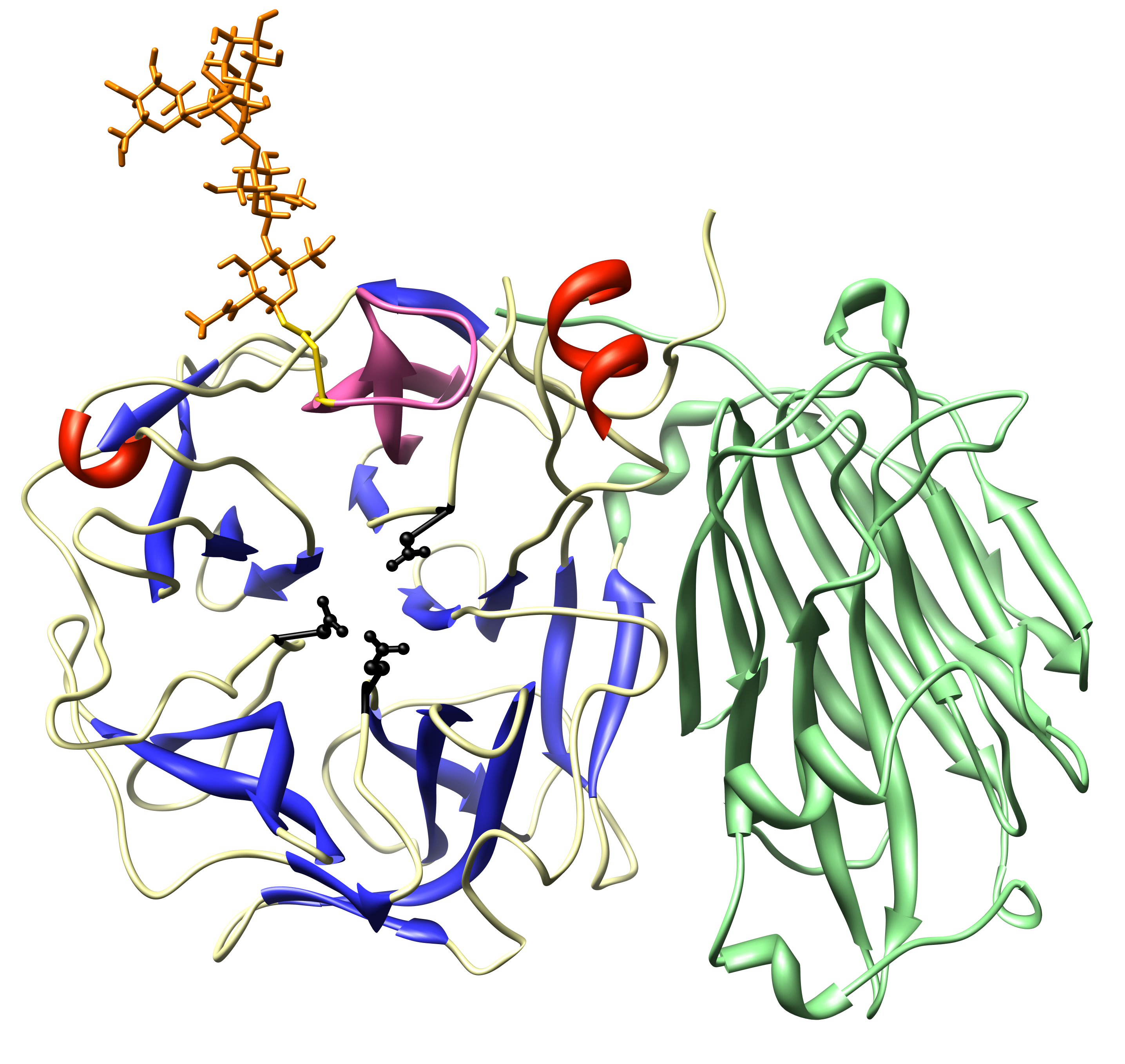

Supplement: Figure S4 — Protein tertiary structure with modeled N-glycans. (TIF) [file pcbi.1002285.s004.tif]
