# Supplementary material for: Integrating Bioinformatics Tools to Handle Glycosylation
Source: PLoS Comput Biol. 2011 Dec 29;7(12):e1002285. doi: 10.1371/journal.pcbi.1002285 (PMC3248387; doi:10.1371/journal.pcbi.1002285)
Supplement: Figure S2 — Multiple sequence alignment. (PDF) [file pcbi.1002285.s002.pdf]

```
Conservation:   0010000011543786560111111264549967443528288397847403311012043978645
SS                                           IA          IB                  IC
TARGET          S-VDKVKQIHRTGYHFQPP------KNWINDPNGPLYYKGLYHLFYQYNPKGA--VWGNIVWAHSVS
Q93X60          LANQQIEQPYRTGYHFQPP------SNWMNDPNGPMLYQGVYHFFYQYNPYAAT-FGDVIIWGHAVS
Q43866          KS-PSVNQPYRTGFHFQPP------KNWMNDPNGPMIYKGIYHLFYQWNPKGA--VWGNIVWAHSTS
A0A7Z0          PWNNSMLSWQRTAFHFQPE------KNWMNDPNGPLFYKGWYHFFYQWNPNGA--VWGDIVWGHAVS
A3QRG0          PWSNAMLQWQRTGFHFQPE------QHYMNDPNGPVYYGGWYHLFYQHNPKGD--SWGNIAWAHAVS
A5GXL9          S---EDLQPYRTAFHFQPL------KNWMNDPNGPMYFNGVYHLFYQYNPGGP--LWGNISWGHSIS
A7IZK7          SP----SQTYRTSYHFQPP------KNWMNDPNGPTVYRGLYHLFYQYNPLGP--DWGNIVWAHSTS
A7IZK8          PWTNAMLSWQRTSYHFQPE------KNWMNDPNGPLFHMGWYHLFYQYNPDSA--IWGNITWGHAVS
A7LJR5          PFTTKMLQWQHTGFHFQPP------RYFMSDPSGPVYYKGWHHFFYQHNAKAA--FWGNIAWGHAAS
A7RDD3          PWTNDMLRWQRTGFHFQPE------KNFQADPNAAMFYKGWYHFFYQYNPTGV-AWDYTISWGHAVS
A9CZQ1          ---PLASTQFRTAYHFQPT------HYWMNDPNAPMYYDGVYHLFYQYNPNGA-TWTAYMSWGHSVS
A9E2W4          ---FIASELYRTAYHFQPT------QHWMNDPNAPMYYNGVYHLFYQHNPDAA-TWTANISWGHSVS
A9JIF3          LPTQIIEQPYRTGYHFQPP------SNWMNDPNGPMLYNGVYHFFYQYNPYAA-TFGDVIVWGHAVS
A9LST6          AWSNAMLSWQRTAYHFQPQ------KNWMNDPNGPLYHKGWYHLFYQYNPDSA--IWGNITWGHAVS
A9YTS8          LESNAEVEWERSAYHFQPD------KNFISDPDGPMYHMGWYHLFYQYNPESA--IWGNITWGHSIS
A9YTS9          YSQSDRLTWERTAYHFQPQ------KNFIYDPNGPLFHMGWYHLFYQYNPYAP--IWGNMSWGHAVT
B0I1Q7          PWSNAMLQWQHTGFHFQPL------KHYMNDPNGPVYYGGWYHLFYQHNPYGD--SWANISWGHAVS
B2NIA0          NWTNAMFSWQRTAFHFQPE------KNWINDPNGPLFHKGWYHLFYQYNPDSA--VWGNITWGHAVS
O04372          PWTNKMLSWQRTGFHFQPE------KNWMNDPNGPLYYKGWYHFFYQYNPNAA--VWGDIAWGHAVS
O23786          LKTNAEVEWQRSAYHFQPD------KNYISDPDGPMYHMGWYHLFYQYNPESA--IWGNITWGHSVS
O24459          LESNADVEWQRSAYHFQPD------KNFISDPDGPMYHMGWYHLFYQYNPESA--IWGNITWGHSVS
O65341          PWSNAMLQWQRTGFHFQPQ------KNWMNDPNGPVYYKGWYHLFYQYNPDGA-IWGNKIAWGHAVS
O65342          PWSNAMLQWQRTGFHFQPQ------KNWMNDPNGPVYYKGWYHLFYQYNPDGA-IWGNKIAWGHAVS
O65778          PSAADRLRWERTAFHFQPA------KNFIYDPNGPLFHMGWYHLFYQYNPYAP--FWGNMTWGHAVS
O81082          PWTNEMLKWQRTGYHFQPP------NHFMADPNAAMYYKGWYHFFYQYNPNGS-AWDYSISWGHAVS
O81083          PWTNQMLSWQRTGFHFQPV------KNWMNDPNGPLYYKGWYHFFYQYNPEGA--VWGNIAWGHAVS
O81118          E-ATEVDKELRTGYHFQPP------KHWINDPNGPMYYKGLYHLFYQYNPKGA--VWGNIIWAHSVS
O81985          YSQTDRLSWERTAFHFQPA------KNFIYDPDGQLFHMGWYHMFYQYNPYAP--VWGNMSWGHSVS
O81986          LDSSAEVEWQRSTYHFQPD------KNFISDPDGPMYHMGWYHLFYQYNPQSA--IWGNITWGHSVS
O82119          TSHVDASKVHRTGYHFQPP------KNWINDPNGPMYYNGVYHLFYQYNPKGA--TWGNIVWAHSVS
Q05G13          PWSNAMLQWQHTGFHFQPL------KHYMNDPNGPVYYGGWYHLFYQHNPYGD--SWGNVSWGHAVS
Q05JI1          NWTNAMFTWQRTSFHFQPE------KNWMNDPNGPLFYKGWYHLFYQYNPDSA--VWGNITWGHAAS
Q05JI2          PWTNNMLSWQRTAFHFQPQ------KNWMNDPNGPVFYKGWYHLFYQYNPNGA--IWGDIVWGHAVS
Q0PCC5          YSQSDRLIWERTAFHFQPV------KNFIYDPNGPLFHMGWYHLFYQYNPYAP--VWGNMSWGHSVS
Q0PCC7          ----------------QPA------KNFIYDPNGQLFYMGWYHLFYQYNPYGP--VWGNMSWGHSVS
Q0PCC8          SSQFDRLLWERTAFHFQPS------KNFIYDPNGQIFHMGWYHLFYQHNPYAP--VWGNMSWGHSVS
Q0PCC9          PSAADRLRWERTAYHYQPA------KNFMYDPNGPIFHMGWYHLFYQYNPYSV--FWGNMTWGHAVS
Q0W9N0          PWTQKMLAWQRTSFHFQPK------KNWMNDPNGPLYYKGWYHLFYQYNPEAA--VWGNIVWGHAVS
Q1KL65          AWSNAMLSWQRTAYHFQPQ------KNWMNDPNGPLYHKGWYHLFYQYNPDSA--IWGNITWGHAVS
Q2UXF7          LVRHGHGVGIRPAYHFLPA------KNWQNDPNGPMYHNGVYHMFYQYNPLGAMWQPGNLSWGHSVS
Q2WEC6          YSPADRLSWERTAFHFQPA------KNMIYDPDGLLFYMGWYHLFYQHNPYAP--VWGNMTWGHAVS
Q2XQ19          PWSNAMLQWQRTGFHFQPQ------KNWMNDPNGPVYYKGWYHLFYQYNPEGA-VWGNKIAWGHAAS
Q2XQ21          P-PSIVDSQLRTGYHFQPL------KNWINDPNAPMYYKGWYHLFYQYNPKGA--VWGNIVWAHSVS
Q3L7K5          SS-LKTHQPYRTGYHFQPR------KNWMNDPNGPMIYKGLYHFFYQYNPHGA--VWGNIVWAHSTS
Q41215          AWSNAMLSWQRTAYHFQPQ------KNWMNDPNGPLYHKGWYHLFYQYNPDSA--IWGNITWGHAVS
Q41604          PWTDAMLEWQRTGFHFQPE------KNWMNDPDGPMFYKGWYHIFYQYNPVSA--VWGNITWGHAVS
Q41606          PWTDAMLQWQRTGFHFQPE------KNWMNDPDGPMFYKGWYHIFYQYNPVSA--VWGNITWGHAVS
Q42691          -----SNQPYRTAYHFQPR------KNWINDPNGPMLFKGIYHLFYQYNPNGVKLR-GPPVWGHSTS
Q42722          PWNSNVLSWQRSSFHFQPN------QNWMNDPNGPLFYKGWYHLFYQYNPDGA-IWGNKIVWGHAVS
Q43172          S-TVDEKNVHRTGYHFQPP------KNWINDPNAPMYYNGVYHLFYQYNPYGS--VWGNIVWAHSVS
Q43799          SSNVKN--VHRTGYHFQPP------KNWINDPNGPMYYNGVYHLFYQYNPKGA--VWGNIVWAHSVS
Q43818          PLSLISVKTWRCKYQLAPMSHEQAVQNKTNDPNGYIYSKGSYSQFLASNLAGS--INGNVPWAIAVS
Q43855          S-APSVNKLHRTGFHFQPN------RNWINDPNGPMYYRGIYHLFYQYNPKGA--VWGNIVWGHSVS
Q43856          EY--STNQPYRTGYHFQPP------KNWINDPNGPLIYKGVYHLFYQYNPKGA--QWGNIVWAHSVS
Q4AEI9          PWSNAMLQWQRTGYHFQPE------KNYQNDPNGPVYYKGWYHFFYQHNPGGT--GWGNISWGHAVS
Q547Q0          AWSNAMLSWQRTAYHFQPQ------KNWMNDPNGPLYHKGWYHLFYQYNPDSA--IWGNITWGHAVS
Q575T1          PWSNAMLQWQRTGFHFQPE------KNWMNDPNGPVYYKGWYHLFYQYNPDGA-IWGNKIAWGHAAS
Q5ZQK6          LPS---DQPYRTGYHFQPP------QNWMNDPNGPMYYKGVYHFFYQYNPNGP-LFGDIMIWGHSVS
Q64GB3          EVPSIVSDRYRTAYHFQPP------KNWMNDPNGPMYYNGIYHLFFQHNPNGP--QWGDIVWGHSVS
Q6F4N3          PWSNAMLQWQHTGFHFQPL------KHYMNDPNGPVYYGGWYHLFYQHNPYGD--SWGNVSWGHAVS
Q6KCH6          PWSNAMLQWQRTGFHFQPE------KNWMNDPNGPVYYKGWYHLFYQYNPDGA-IWGNKIAWGHAAS
Q6PVN1          PWSNAMLQWQRTGFHFQPE------KNWMNDPNGPVYYRGWYHLFYQYNPEGA--VWGNIAWGHAVF
Q70AT7          QDPSPASTMYKTAFHFQPA------KNWMNDPSGPMYFNGIYHEFYQYNLNGP--IFGDIVWGHSVS
Q70LF5          PWSNAMLQWQRTGFHFQPD------KYYQNDPNGPVYYGGWYHFFYQYNPSGS-VWEPQIVWGHAVS
Q70XE6          HQMINDDDPYRTAYHFQSP------KNWMNDPNGPMIYKGIYHLFYQYYPYDPV-WHTEIVWGHSTS
Q7DLY6          PWNNTILSWQRTAFHFQPE------KNWMNDPNGPLFYKGWYHFFYQYNPNAA--VWGDIVWGHAVS
Q7XA49          ----SSNQPYRTAYHFQPP------KNWINDPNGPLRYAGLYHLFYQYNPKGA--VWGNIVWAHSVS
Q7XAS5          AWSNAMLSWQRTAYHFQPQ------KNWMNDPNGPLYHKGWYHLFYQYNPDSA--IWGNITWGHAVS
Q7XZS5          PWSNAMLQWQRTGFHFQPQ------RNWMNDPNGPVYYKGWYHLFYQYNPDGA-IWGNKIAWGHAVS
```

```
Q84LA1          PSP-AVSTMYKTAFHFQPA------KNWMNDPSGPMYFNGIYHEFYQYNLNGP--IFGDIVWGHSVS
Q84RM0          PWSNAMLQWQRTGFHFQPE------QHYMNDPNGPVYYGGWYHLFYQHNPKGD--SWGNIAWAHAVS
Q84V21          S-AISVKNVHRTRFHFQPP------KHWINDPNAPMYYNGVYHLFYQYNPKGS--VWGNIIWAHSVS
Q84XV1          P-ASVVDNRLRTGYHFQPP------RNWINDPNGPMYFNGVYHLFYQYNPNGS--VWGNIVWAHSVS
Q8GT50          ----FSTPSLSTGYHFRPI------KNWINDPNAPMYYKGWYHLFYQYNPKGA--VWGNIVWAHSVS
Q8GT63          VTTAG-GVRTRSAYHFQPA------KNWQNDPNGPMYHNGLYHFFYQYNPDGVTWGNGNLSWGHSVS
Q8GUA3          PWTNAMLTWQRTAYHFQPQ------KNWMNDPNGPLYHKGWYHLFYQYNPDSA--IWGNITWGHAIS
Q8GUB8          --SNAMLQWQRTGFHFQPE------MNWMNDPNGPVYYRGWYHLFYQYNPEGA--VWGNIAWGHAVS
Q8L6W0          GASAGTTQPYRTAYHFQPL------KNWMNDPNGPLYYKGVYHLFYQYNPYSA--IWGNMTWGHSIS
Q8L6W1          -----TNQPYRTAYHFQPR------KNWINDPNGPMLYKGVYHLFYQYNPNGV--IWGPPVWGHSTS
Q8L6W2          DWNDLQLAWQRTAFHFQPQ------KNWMNDPNGPLYYNGWYHFFYQYNPAGA--VWGNIVWGHAVS
Q8L897          PWDNTMLSWQRTAFHFQPE------KNWMNDPNGPLYYKGWYHFFYQYNPNGA--VWGDIVWGHAVS
Q8LPM7          PWTNDMFEWQRPGYHFQPP------YHFMGDPNAAMYYKGWYHFFYQYNPYGS-AWDTTISWAHAVS
Q8LRN6          --------VHRTRYHFQPP------KNWINDPNAPMYYNGVYHLFYQYNPYGS--VWGNIVWAHSVS
Q8RVH4          PWSNNMLDWQRTAFHFQPK------KNWMNDPNGPVFYNGWYHLFYQYHPDAP--VWGKIVWGHAVS
Q8VXS5          -----TNQPYRTAYHFQPR------KNWINDPNGPMLYKGVYHLFYQDNPNG--VIWGPPVWGHSPS
Q8VXS7          DWNDLQLAWQRTAFHFQPQ------KNWMNDPNGPLYYNGWYHFFYQYNPAGA--VWGNIVWGHAVS
Q8W3M2          PWTNNMLSWQRTAFHFQPQ------KNWMNDPNGPVFYKGWYHLFYQYNPNG--AIWGDIVWGHAVS
Q93X59          LANQQIQQPYRTGYHFQPP------SNWMNDPNGPMLYQGVYHFFYQYNPYAATFG-DVIIWAHAVS
Q941I4          NWTNAMLSWQRTAFHFQPE------RNWMNDPDGPMFYKAWYHLFYQYNPNSA--LWGNITWGHAVS
Q944C8          NWTNAMFTWQRTSFHFQPE------KNWMNDPNGPLFYKGWYHLFYQYNPDSA--VWGNITWGHAVS
Q944U7          S-VDIVDQIHRTAYHFQPP------KHWINDPNAPMYYNGVYHLFYQYNPKGA--VWGNIVWAHSVS
Q94C05          PWSNLMLTWQRTAYHFQPE------KNWMNDPDGPLYHKGWYHFFYQYNPDSA--VWGNITWGHAVS
Q94C06          AWSNLMLSWQRTSYHFQPV------KNWMNDPNGPLYYKGWYHLFYQYNPDSA--VWGNITWGHAVS
Q94C07          AWTNIMLTWQRTTYHFQPA------QNWMNDPNGPLYYKGWYHLFYQWNPDTA-VWGNKISWGHAV-
Q94C08          VWSNVMLVWQRTVFHFQPE------KNWMNDPNGPLYHKGWYHIFYQWNPEGA-AWGNKISWGHAVD
Q9AUH1          PWSNEMLQWQRSSYHFQPA------KNYMSDPDGLLYYGGWYHMFYQYNPVGT-DWADGMAWGHAVS
Q9FNS9          SSPTEESQPYRTGFHFQPP------KNWINDPNGPMYFNGVYHLFYQYNPYGP--LWGNISWGHSIS
Q9FQ62          AWNNSVLSWQRTAFHFQPE------QNWMNDPNGPLFYKGWYHFFYQYNPNAA--VWGDIVWGHAVS
Q9FR47          PWSNSMLQWQRAGFHFQTE------KNFMSDPNGPVYYRGYYHLFYQYNTKGV-VWDDGIVWGHVVS
Q9LD97          S-AISVKNVHRTRFHFQPP------KHWINDPNAPMYYNGVYHLFYQYNPKGS--VWGNIIWAHSVS
Q9LDS8          S-AISVKNVHRTRFHFQPP------KHWINDPNAPMYYNGVYHLFYQYNPKGS--VWGNIIWAHSVS
Q9M4K8          R-AISVKNVHRTGFHFQPP------KYWINDPNAPMYYNGVYHLFYQYNPKGS--VWGNIVWAHSVS
Q9SBI2          P-PSIVNPLLRTGYHFQPP------KNWINDPNAPMYYKGWYHFFYQYNPKGA--VWGNIVWAHSVS
Q9SM30          LESNAGVEWERSAYHFQPD------KNFISDPDGPMYHMGWYHLFYQYNPESA--IWGNITWGHSIS
Q9SPK0          P-PSIVNPLLRTGYHFQPP------KNWINDPNAPMYYKGWYHFFYQYNPKGA--VWGNIVWAHSVS
Q9ZP42          STHPQAKDPYRTGYHFQPR------KNWINDPNGPLIYKGIYHLFYQYNPSSV--VWGNIVWAHSTS
Q9ZR55          PATS--EQPYRTAFHFQPP------QNWMNDPNGPMCYNGVYHLFYQYNPFGP-LWNLRMYWAHSVS
Q9ZR96          HYQSDRLTWERTAYHFQPA------KNFIYDPNGPLFHMGWYHLFYQYNPYAP--IWGNMSWGHAVS
Q9ZTW9          AWSNLMLSWQRTSYHFQPV------KNWMNDPNGPLYYKGWYHLFYQYNPDSA--VWGNITWGHAVS
Q9ZTX2          PWSNLMLTWQRTAYHFQPE------KNWMNDPNGPLYYKGWYHFFYQYNPDSA--VWGNITWGHAVF
P92916          PWTNDMLAWQRCGFHFRTV------RNYMNDPSGPMYYKGWYHLFYQHNKDFA--YWGNITWGHAVS
Q5FC15          AWTNQMLTWQRAGFHFRTV------KNYMNDPSGPMYYKGWYHLFYQHNPNYA--YWGDISWGHAVS
P93761          TWSNAMLNWQRTAYHFQPQ------KNWMNDPNGPLYHKGWYHLFYQYNPDSA--IWGNITWGHAVS
P49175          SWTNAMLAWQRTAFHFQPP------KNWMNDPNGPLYHKGWYHLFYQWNPDSA--VWGNITWGHAVS
Q43089          HSSKSNHQPYRTAYHFQPL------KNWINDPNGPMRYGGFYHLFYQYNPKGA--VWGNIVWAHSVS
Q39692          S-AVNVQLVHRTGYHFQPK------KHWINDPNGPMYYKGFYHLFYQYNPKGA--VWGNIVWAHSIS
Q39693          S-AVDVKLVHRTGYHFQPQ------KHWINDPNGPMFYKGYYHLFYQYNPKGS--VWGNIVWAHSVS
Q56UD0          ANGHR----DRTAYHFQPA------KNWQNDPNGPVYYNGMYHLFYQYNPHGALWDVGNLSWGHSVS
Q0J360          ANHAR-----RTAYHFQPA------KNWQNDPNGPMYHNGMYHLFYQYNPHSALWDIGNLSWGHSVS
P29001          PWDNSMLSWQRTSFHFQPE------KNWMNDPNGPMYYKGWYHFFYQYNPNGA--VWGDIVWGHAVS
O24509          PWDNSMLSWQRTSFHFQPE------KNWMNDPNGPMYYKGWYHFFYQYNPNGA--VWGDIVWGHAVS
P29000          AWSNAMLSWQRTAYHFQPQ------KNWMNDPNGPLYHKGWYHLFYQYNPDSA--IWGNITWGHAVS
Q43857          PWNNTMLSWQRTAFHFQPE------KNWMNDPNGPLYYKGWYHFFYQYNPNGA--VWGDIVWGHAVS
P80065          PWTNDMLSWQRTSFHFQPQ------ENWMNDPNGPLFHMGWYHLFYQYNPDSA--IWGNITWGHAIS


Conservation:   119775801641740731346019448883736443115445899120111111233
SS                       ID                  IIA           IIB
TARGET          -KDLINWESLEPAIYPSKWFDNYGCWSGSATILPNG-EPVIFYTGIVDGNNR------
Q93X60          -YDLVNWIHLDPAIYPTQEADSKSCWSGSATILPGN-IPAMLYTGSDSKSR-------
Q43866          -TDLINWDPHPPAIFPSAPFDINGCWSGSATILPNG-KPVILYTGIDPKNQ-------
A0A7Z0          -KDLIHWLHLPLAMVADQWYDINGVWTGSATILPDG-KIVMLYTGST-------NESV
A3QRG0          -KDMVNWRHLPLAMVPDQWYDSNGVLTGSITVLPDG-QVILLYTGNTDT-------LA
A5GXL9          -HDLVNWFILEPALSPKEPYDIGGCFTGSSTILHGS-KPIILYTAQDVDGA-------
A7IZK7          -KDLINWNPHKAAIFPSQKGDVNGCWSGSTTMLRGE-NPAILYTGIDPKSQ-------
A7IZK8          -RDLIHWLYLPFAMVPDRPFDINGVWTGSATILPGG-QIVILYTGDT-------ADLV
A7LJR5          -RDLLNWVHLPLAVEPDHWYDIEGDWTGSVAVLPDG-RVIMLFTGGTGA-----NELA
A7RDD3          -KDLLHWNYLPMALRPDHWYDRKGVWSGYSTLLPDG-RIVVLYTGGT-------KELV
A9CZQ1          -TDLVHWTGLELALTPSDPFDISGCWSGSATILPGN-KPVVLYTG-LDTVGR------
A9E2W4          -ADLVHWTGLELALTPSDPFDISGCWSGSATILPGS-KPVILYTG-LDTVSR------
```

A9JIF3	-YDLVNWIHLDPAIYPTHEADAKSCWSGSATILPGN-IPAMLYTGSDSHSR-------
A9LST6	-KDLIHWLYLPFAMVPDQWYDINGVWTGSATILPDG-QIMMLYTGDT-------DDYV
A9YTS8	-RDMINWFHLPFAMVPDHWYDIEGVMTGSATVLPNG-QVIMLYTGNAY-------DLS
A9YTS9	-KDMINWFELPVALTPTEWYDFEGVLSGSTTALPNG-QIFALYTGNAN-------DFS
B0I1Q7	-KDMVNWRHLPVALVPDQWYDINGVLTGSITVLPDG-RVILLYTGNTDT-------FA
B2NIA0	-TDMIHWLYLPLAMVPDRWFDANGVWTGSATLLPDG-QIIMLYTGST-------NESV
O04372	-KDLLSWRHLPLAMVPDRWYDINGVWTGSATILPDG-RIIMLYTGAT-------NESV
O23786	-KDMINWFHLPFAMVPDQWYDIEGVMTGSATVLPDG-QIIMLYTGNAY-------DLS
O24459	-RDMINWFHLPFAMVPDHWYDIEGVMTGSATVLPNG-QIIMLYTGNAY-------DLS
O65341	-RDLIHRRHLPLAMVPDQWYDTNGVWTGSATTLPDG-RLAMLYTGST-------NASV
O65342	-RDLIHRRHLPLAMVPDQWYDTNGVWTGSATTLPDG-RLAMLYTGST-------NASV
O65778	-KDMINWFELPIALAPTEWYDIEGVLSGSTTILPDG-RIFALYTGNTN-------DLE
O81082	-KDMIHWLHLPVAMVPDHWYDSKGVWSGYATTLPDG-RIIVLYTGGT-------DQLV
O81083	-RDLVHWTHLPLAMVPDQWYDINGVWTGSATILPDG-QIVMLYTGAT-------NESV
O81118	-TDLVDWVALEPGIYPSKPFDINGCWSGSATILPNG-VPVIMYTGIEPKET-------
O81985	-KDMINWYELPVAMVPTEWYDIEGVLSGSTTVLPNG-QIFALYTGNAN-------DFS
O81986	-KDMINWFHLPFAMVPDHWYDIEGVMTGSATVLPNG-QIIMLYSGNAY-------DLS
O82119	-KDLINWIPLEPAIYPSKVFDKYGTWSGSATILPGN-KPVILYTGIVDA-----NKT-
Q05G13	-KDLVNWRHLPVALVPDQWYDINGVLTGSITVLPDG-RVILLYTGNTDT-------FS
Q05JI1	-TDLIHWLYLPIAMVPDQWYDINGVWTGSATILPDG-QIVMLYTGST-------DKSV
Q05JI2	-KDLIHWYHLPLAMVADQWYDIMGVWTGSATILPDG-KLMMLYTGST-------NESV
Q0PCC5	-KDLVNWLELPVALTPTEWYDIEGVLSGSTTALPNG-QIFALYTGNAN-------DFS
Q0PCC7	-KDMIHWFELPVALTPSKWYDSEGVLSGSITVLPNG-EIFALYTGNAN-------DFS
Q0PCC8	-KDMINWFELPVAMIPTEWYDIEGVLSGSITVLPNG-QIFALYTGNAN-------DFS
Q0PCC9	-KDMINWFELPVALAPVEWYDIEGVLSGSTTVLPTG-EIFALYTGNAN-------DFS
Q0W9N0	-RDLIHWQHLPVAMVADQWYDINGVWTGSATFLPNG-DLIMLYTGST-------NESI
Q1KL65	-KDLIHWLYLPFAMVPDQWYDINGVWTGSATILPDG-QIMMLYTGDT-------DDYV
Q2UXF7	-RDLVNWDALDTALDPTAPFDYNGCWSGSATILPGG-IPALLYTGRIDADKEV-----
Q2WEC6	-KDMVNWYELPIAMVPTEWYDIEGVLSGSITVLPNG-KIFALYTGNAN-------DFS
Q2XQ19	-RDLLHWRHLPIAMLPDRWYDINGVWTGSATTLPDG-RLAVLYTGST-------NTSV
Q2XQ21	-RDLINWVSLKPAIEPSIKSDKYGCWSGSATMTLDG-TPAIMYTGVNRPDVNY-----
Q3L7K5	-TDLVNWTPHKYAISPSQPADINGCWSGSATILPNG-KPVILYTGIDPQNK-------
Q41215	-KDLIHWLYLPFAMVPDQWYDINGVWTGSATILPDG-QIMMLYTGDT-------DDYV
Q41604	-RNLIHWFHLPIAFVPDQWYDANGALTGSATFLPDG-RIAMLYTGITTEF-------V
Q41606	-RNLIHWFHLPIAFFPDQWYDARGALTGSATFLPDG-SIAMLYTGITTEF-------V
Q42691	-KDLVNWMPQPLTMEPEMAANINGSWSGSATILPGN-KPAILFTGLDPNYE-------
Q42722	-SDLIHWKHLPVAMVTDHWYDVNGVWTGSATILPDG-QIVMLYTGST-------NESV
Q43172	-TDLINWIPLEPGIYPSEVFDKYGTWSGSATILPNN-KPIILYTGIVDAKNT------
Q43799	-TDLINWIPLEPAIYPSKIFDKFGTWSGSATILPGN-KPIILYTGIIDANRT------
Q43818	-LDEINWIVLNFAISRDQPIYINGIVSTSELILDGN-LPVITGTAIDEN--------V
Q43855	-KDLINWKELEPALFPSKPFDKYGCWSGSATILPGK-GPVILYTGVVDKQSN------
Q43856	-TDLINWIPLDPAIFPSQPSDINGCWSGSTTILHGN-KPSILYTGINKLNH-------
Q4AEI9	-RDMVHWRHLPLAMVPEHWYDIEGVLTGSITVLPDS-RVILLYTGNTET-------FA
Q547Q0	-KDLIHWLYLPFAMVPDQWYDINGVWTGSATILPDG-QIMMLYTGDT-------DDYV
Q575T1	-RDLLRWRHLPVAMSPDQWYDINGVWSGSATVLPDG-RIVMLYTGST-------NASV
Q5ZQK6	-YDLVNWIHIDPAIYPTDPADINSCFSGSATFLPGY-KPVMLYTGLDTEKR-------
Q64GB3	-TDLVNWIILEPAIEPDTPGDIKGCWSGSATILFGG-QLVIMYTG-GDVENH------
Q6F4N3	-KDLVNWRHLPVALVPDQWYDINGVLTGSITVLPDG-RVILLYTGNTDT-------FS
Q6KCH6	-RDLLRWRHLPVAMSPDQWYDINGVWSGSATVLPDG-RIVMLYTGST-------NASV
Q6PVN1	-RDLIHWRHLPLAMVPDQWYDINGVWTGSATVLPDG-SLVMLYTGST-------NASV
Q70AT7	-TDLVNWIGLEPALVRDTPSDIDGCWTGSVTILPGG-KPVIIYTG-GNIDQH------
Q70LF5	-KDLIHWRHLPPALVPDQWYDIKGVLTGSITVLPDG-KVIPLYTGNTET-------FA
Q70XE6	-TDLINWTQQPIALSPSEPYDINGCWSGSITILPQN-KPVILYTGINNKNY-------
Q7DLY6	-KDLIHWLYLPIAMVPDQWYDANGVWTGSATFLDDG-SIVMLYTGST-------DEFV
Q7XA49	-KDLVNWTPLDPAIFPSQPSDINGCWSGSTTLLPGN-KPVILYTGIDLLNQ-------
Q7XAS5	-KDLIHWLYLPFAMVPDQWYDINGVWTGSATILPDG-QIMMLYTGDT-------DDYV
Q7XZS5	-RDLIHWRHLPLAMLPDQWYDTNGVWTGSATTLPDG-RLAMLYTGST-------NASV
Q84LA1	-TDLVNWIGLEPALVRDTPSDIDGCWTGSVTILPGG-KPIIIYTG-GDIDQH------
Q84RM0	-KDMVNWRHLPLAMVPDQWYDSNGVLTGSITVLPDG-QVILLYTGNTDT-------LA
Q84V21	-KDLINWIHLEPAIYPSKKFDKYGTWSGSSTILPNN-KPVIIYTGVVDSYNN------
Q84XV1	-TDLVNWIALDPAIRPSKPFDINGCWSGSATVLPGN-RPVIVYTGIDPQQR-------
Q8GT50	-RDLINWVALETAIQPSIKSDKYGCWSGSAXILRDG-TPAIMYTGIDRADINY-----
Q8GT63	-VDLVNWFALDAALQPSRPFDANGCWSGSATILPDG-SPVMLYTG-IDARGD------
Q8GUA3	-TDLIHWLYLPFALVPDQWYDINGVWTGSATFLPDG-QIMMLYTGDT-------NDYV
Q8GUB8	-RDLVHWRHLPLAMVPDQWYDINGVWTGSATVFPDG-TLNMLYTGST-------NASV
Q8L6W0	-NDLVNWVHLEHALNPIEPYELGGCFSGSITMLPGG-RPVIFYTGADTNNF-------
Q8L6W1	-KDLVNWVPQPLTMEPEMAANINGSWSGSATILPGN-KPAILFTGLDPKYE-------
Q8L6W2	-KDLIRWKHLPIAMVADRWYDFNGVWTGSATILPDG-QIMMLYTGST-------NESV
Q8L897	-RDIIHWLHLPLAMVADQWYDSNGVWTGSATILPDG-QVIMLYTGST-------NESV
Q8LPM7	-KDMIHWLHLPIAMVPDHWYDSKGVWSGYATTLPDG-RIIVLYTGGT-------DELV
Q8LRN6	-TDLINWIPLEPAIYPSKVFDKYGTWSGSATILPDN-KPIILYTGIVDAKNT------

```
Q8RVH4          -KDLINWRHLPIAMETDEWYDEQGVWTGSATILPNG-ELVVLYTGST-------NESV
Q8VXS5          -KDLVNWVPQPLTMEPEMAANINGSWSGSATILPGN-KPAILFTGLDPKYE-------
Q8VXS7          -KDLIRWKHLPIAMVADRWYDFNGVWTGSATILPDG-QIMMLYTGST-------NESV
Q8W3M2          -KDLIHWYHLPLAMVADQWYDIMGVWTGSATILPDG-KLMMLYTGST-------NESV
Q93X59          -YDLVNWIHLDPAIYPTQEADSKSCWSGSATILPGN-IPAMLYTGSDSKSR-------
Q941I4          -PDLIHGSTSRWLWSPIDGSMPTGCG-WVCAILPDG-QIAILYTAST-------NESV
Q944C8          -ADLIHWLYLPIAMVPDQWYDINGVWTGSATILPDG-QIVMLYTGST-------DKSV
Q944U7          -TDLINWIPLKPAIVPSEPFDIKGCWSGSATVLPNN-IPIILYTG-LDS-----NET-
Q94C05          -RDLIHWFHLPFAMVPDQWYDINGVWTGSATILPDG-QIVMLYTGST-------DENV
Q94C06          -TDLINWLHLPFAMVPDQWYDVNGVWTGSATILPDG-RIVMLYTGDT-------DDYV
Q94C07          SKDLLHWHHLPIAMVPDNWYDLNGVWSGSATDLPDG-KLMMLYTGSTV------DQSV
Q94C08          SKDLLHWYHLPWAMYPDQPYDLNGVWTGSATTLPDG-KLAMLYTGETE------DELV
Q9AUH1          -RNLVQWRTLPIAMKPDQWYDILGVLSGSVTVLPNG-TVIMLYTGATN------DWYV
Q9FNS9          -YDLVNWFLLEPALSPKEPYDINGCLSGSATILPGP-RPIILYTGQDV------NNS-
Q9FQ62          -KDLIHWVHLPIAMVADQWYDANGVWTGSATFLDDG-SLVMLYTGST-------DKSV
Q9FR47          -RDLVHWRHLPIAMVPDHWYDNMGVLSGSITVLNSG-RLVMIYTGVFSNTTDR-SGMM
Q9LD97          -KDLINWIHLEPAIYPSKKFDKYGTWSGSSTILPNN-KPVIIYTGVVDSYNN------
Q9LDS8          -KDLINWIHLEPAIYPSKKFDKYGTWSGSSTILPNN-KPVIIYTGVVDSYNN------
Q9M4K8          -KDLINWIHLEPAIYPSKKFDKYGAWSGSATILPNN-KPVILYTGVVDSHDS------
Q9SBI2          -RDLINWVALEPALRPSIPGDRYGCWSGSATVLPDGGGPVIMYTGVDHPDINY-----
Q9SM30          -RDMINWFHLPFAMVPDHWYDIEGVMTGSATMLPDG-QIIMLYTGNAY-------DLA
Q9SPK0          -RDLINWVALEPALRPSIPGDRYGCWSGSATVLPDGGGPVIMYTGVDHPDINY-----
Q9ZP42          -TDLVNWIPHEAAIYPSILSDINGCWSGSVTILPSG-KPAILYTGINPDKE-------
Q9ZR55          -HDLINWIHLDLAFAPTEPFDINGCLSGSATVLPGN-KPIMLYTGIDTENR-------
Q9ZR96          -KDMINWFELPVALTPTEWYDIEGVLSGSTTALPNG-QIFALYTGNAN-------DFS
Q9ZTW9          -TDLINWLHLPFAMVPDQWYDVNGVWTGSATILPDG-RIVMLYTGDT-------DDYV
Q9ZTX2          PTDLINWIHLPFAMVPDQWYDVNGVWTGSATILPDG-RIVMLYTGDT-------DDYV
P92916          -RDLINWQHLPVAVGPDHWYDISGVWTGSIIVVSED-RVVMLFTGGT-------KSFD
Q5FC15          -RDLLNWFHLPVAVKPDRWYDIYGVWTGSITVMPDDGRVVMLYTGGT-------KEKY
P93761          -TDLIHWLYLPFAMVPDQWYDINGVWTGSATILPDG-LIMMLYTGDT-------DDYV
P49175          -RDLLHWLHLPLAMVPDHPYDANGVWSGSATRLPDG-RIVMLYTGSTAESS------A
Q43089          -KDLVNWTPLDHAIHPSQPSDIKGCWSGSATILPGG-KPAILYTGIDPNNH-------
Q39692          -KDLINWVALEPAIFPSKPFDKYGCWSGSATVLPGG-KPVIMYTGIVTPSPVNT----
Q39693          -KDLINWIALEPAIFPSKPFDQYGCWSGSATILPGN-KPVILYTGIVSPDPENA----
Q56UD0          -GDLVNWAALDNALDPTAPFDANGCASGSVTILPDG-VPVVMYSG-IDARRR------
Q0J360          -GDLLNWAALDTALDPTSPFDANGCWSGSATILPGA-LPAILYTG-IDASKE------
P29001          -RDMIHWLHLPLAMVADQWYDKQGVWTGSATILPNG-EIIMLYTGST-------NESV
O24509          -RDMIHWLHLPLAMVADQWYDKQGVWTGSATILPNG-EIIMLYTGST-------NESV
P29000          -KDLIHWLYLPFAMVPDQWYDINGVWTGSATILPDG-QIMMLYTGDT-------DDYV
Q43857          -RDLIHWLHLPLAMVADQWYDSNGVWTGSATILPDG-QVIMLYTGST-------NEFV
P80065          -RDLINWLHLPFAMQPDQWYDINGVWTGSATVLPDG-KIVMLYTGDT-------DDLV

Conservation:   734427163503654923927112199640320031111101289884726020311320
SS                 IIC            IID                        IIIA
TARGET          QIQNYAVPANSSDPYLREWVKPD-DNPIVYPDPSV----NASAFRDPTTAWR-VG---GH
Q93X60          QVQDLAWPKNLSDPFLREWVKHP-KNPLITPPEGVK----DDCFRDPSTAWLGPD---GV
Q43866          QVQNIAEPKNLSDPYLREWKKSP-LNPLMAPDAVNG--INASSFRDPTTAWLGQD---KK
A0A7Z0          QVQNLAYPADHNDPLLTKWVKYSG-NPILVPPPGIG----YKDFRDPTTAWYT---SQGK
A3QRG0          QVQCLATPADPSDPLLREWVKHPA-NPILYPPPGIG----LKDFRDPLTAWFDHS--DHT
A5GXL9          QVQNLALPKNRSDPLLKDWIKWS-GNPILTPVNDI----NTSQFRDPSTAWMGPD---GK
A7IZK7          QVQNLAVPRNLSDPYLIEWVKSP-YNPLMTPTPENK--IDSSSFRDPTTAWLGPD---GR
A7IZK8          QVQNLAYPANLSDPLLLDWIKYPG-NPVMIPPPGIG----KKDFRDPTTAWLA--PDGTK
A7LJR5          QVVNLAVAADPSDPLLMEWIKYDA-NPVLHPPRGIG----LKDFRDPNPVWYNSS--EST
A7RDD3          QVQNLAVPVNLSDPLLLEWKKSHV-NPILVPPPGIE----DHDFRDPFPVWYNES--DSR
A9CZQ1          QVQNIAYPKNLSDPFLREWIKPN-YNPVIEPHQK-IN---AALFRDPSTAWLGKD---GS
A9E2W4          QVQNIAYPKNLSDPFLREWIKPR-YNPVIEPHGR-ID---AALFRDPSTAWLGRD---GS
A9JIF3          QVQDLAWPKNRSDPFLREWVKYT-GNPLITAPEGVN----DDCFRDPSTAWQGPD---GV
A9LST6          QVQNLAYPANLSDPLLLDWVKFKG-NPVLVPPPGIG----VKDFRDPTTAWTG--PQNGQ
A9YTS8          QLQCLAYAVNSSDPLLLEWKKYEG-NPILFPPPGVG----YKDFRDPSTLWMGP---DGE
A9YTS9          QLQCKAVPVNMSDPLLVEWVKYED-NPILYTPPGIG----LKDYRDPSTVWTGP---DGK
B0I1Q7          QVQCLAVPADPSDPLLRSWIKHPA-NPILFPPPGIG----LKDFRDPLTAWFEHS--DKT
B2NIA0          QVQNLAYPANLSDPLLLDWVKYEG-NPILTPPSGIG----STDFRDPTTAWIG---PDGK
O04372          QVQNLAVPADLSDPLLLEWTKVDDANPILVPPPGVG----ATDFRDPTTAWFEPS--DST
O23786          QLQCLAYAVNSSDPLLLDWKKYEG-NPILFPPPGVG----YKDFRDPSTLWLGP---DGE
O24459          QLQCLAYAVNSSDPLLLEWKKYEG-NPILFPPPGVG----YKDFRDPSTLWMGP---DGE
O65341          QVQCLAVPADDADPLLTNWTKYEG-NPVLYPPPGIG----PRDFRDPTTAWFDPS--DST
O65342          QVQCLAVPADDADPLLTNWTKYEG-NPVLYPPPGIG----PRDFRDPTTAWFDPS--DST
O65778          QLQCKAVPVNASDPLLVEWVRYDA-NPILYAPSGIG----LTDYRDPSTVWTGP---DGK
O81082          QVQNLAEPADPSDPLLIEWKKSNG-NPILMPPPGVG----PHDFRDPFPVWYNES--DST
O81083          QVQNLAVPADQSDTLLLRWKKSE-ANPILVPPPGIG----DKDFRDPTTAWYEPS--DDT
O81118          PSAERRVPGQPLRPFLRKWVKPD-YNPIINPDHGI----NASAFRDPTTAWYGPD---GH
```

```
O81985          QLQCKAVPVNLSDPLLIEWVKYED-NPILYTPPGIG----LKDYRDPSTVWTGP---DGK
O81986          QVQCLAYAVNSSDPLLIEWKKYEG-NPVLLPPPGVG----YKDFRDPSTLWSGP---DGE
O82119          QVQNYAIPANMSDPYLRKWIKPD-NNPLIVADKNIN----KIQFRDPTTAWMGRD---GY
Q05G13          QVQCLAVPADPSDPLLRSWIKHPA-NPILFPPPGIG----LKDFRDPLTAWFEHS--DNT
Q05JI1          QVQNLAYPADPSDPLLLDWVKYPG-NPVLVPPRHIG----PKDFRDPTTAWAG---PDGK
Q05JI2          QVQNLAYPADPSDPLLIKWVKYPG-NPVLVPPPGIG----AKDFRDPTTAWLT---SEGK
Q0PCC5          QLQCKAVPVNTSDPLLVEWVRIDS-NPILYTPPGIG----LKDYRDPSTVWTGP---DGK
Q0PCC7          QLQCKAVAVNLSGPLLVEWVRYED-NPILYTPPGIG----LKDYRNPSTVWTGP---DGK
Q0PCC8          QLQCKAIPVNTSDPLLVKWVKYDS-NPILYTPPGIG----LKDYRDPSTVWTGP---DGI
Q0PCC9          QLQCKAVPVNTSDPLLIDWVRYEG-NPILYTPPGVG----LTDYRDPSTVWTGP---DNI
Q0W9N0          QVQNLAYPADPSDPLLRKWIKYEG-NPVPIPPPGIG----LKDFRDPTTAWTT---PEGK
Q1KL65          QVQNLAYPTNLSDPLLLDWVKYKG-NPVLVPPPGIG----VKDFRDPTTAWTG--PQNGQ
Q2UXF7          QVQNVAFPKNPADPLLREWVKPA-YNPVI-PLPADVP---GDNFRDPTTAWVGRD---GL
Q2WEC6          QLQCKAVPVNLSDPLLIKWVKYDD-NPILYTPPGIG----LKDYRDPSTVWTGP---DGK
Q2XQ19          QVQCLALPTNPEDPLLTNWIKYEG-NPVLYPPPAIG----AKDFRDPTTAWLDPS--DKT
Q2XQ21          QVQNVAFPKNKSDPLLQEWDKPG-HNPVIVPEGGI----NATQFRDPTTAWHADG----H
Q3L7K5          QVQNMAVPKNLSDPFLLEWTKLS-QNPLMEPTTINS--INASSFRDPTTAWQGTD---GR
Q41215          QVQNLAYPANLSDPLLLDWVKLKG-NPVLVPPPGIG----VKDFRDPTTRWTG--PQNGQ
Q41604          QVQCQVYPEDVDDPLLLKWFKSDA-NPILVPPPGIG----SKDFRDPTTAWYDVA--EAS
Q41606          QVQCQVYPEDVDDPLLLKWYKSDA-NPILVPPPGIG----SKDFRDPTTAWYDVA--EAS
Q42691          QVQVLAYPKDLNDPYLKEWFLAP-KNPVMFPTPQNQ--INATSYRDPTTAWMLPD---GN
Q42722          QVQNLAYPADPSDPLLIEWVKYPG-NPVLVPPPGID----FKDFRDPTTAWRT---PEGK
Q43172          QVQNYAIPANISDPFLRKWIKPD-NNPLIVADVSI----NKTQFRDPTTCWLGQD---GY
Q43799          QVQNYAIPANLSDPYLRKWIKPD-NNPLIVADMSI----NKTQFRDPTTAWMGRD---GH
Q43818          QVINIAKNRNLSDNYLLDWSKVKG---IPLGVINVGT----NKFRDSTGAWNPKI---GH
Q43855          EVQCIAIPANASVPLLTNWGKPDRLNPILTADHRM----NGSVFRDPTTAWFGKD---GH
Q43856          QVQNLAYPKDFSDPFLREWIKSP-ENPVIEPTSENK--INASSFRDPTTGWLGKD---GK
Q4AEI9          QVTCLAEAADPSDPLLREWVKHPA-NPVVYPPPGIG----MKDYRDPTTAWFDNS--DNT
Q547Q0          QVQNLAYPANLSDPLLLDWVKFKG-NPVLVPPPGIG----VKDFRDPTTAWTG--PQNGQ
Q575T1          QVQCLAFPTDPSDPLLINWTKYEN-NPVMYPPPGVG----EKDFRDPTTAWFDGS--DDT
Q5ZQK6          QVQNLAVPKNLSDPFLREWVKHK-ANPIMTTPEGVK----ADDFRDPSTAWLGYD---GK
Q64GB3          QVQNIALPKNRSDLYLREWTKAG-NNPVLQPVGPGMN---PGEFRDPTTGWIGPD---GL
Q6F4N3          QVQCLAVPADPFDPLLRSWIKHPA-NPILFPPPGIG----LKDFRDPLTAWFEHS--DNT
Q6KCH6          QVQCLAFPTDPSDPLLINWTKYEN-NPVMYPPPGVG----EKDFRDPTTAWFDGP--DDM
Q6PVN1          QVQCLAVPADPNDSLLRNWTKYEA-NPILVPPPGIG----DKDFRDPTTAWFDES--DKT
Q70AT7          QTQNIAFPKNRSDPYLREWIKAA-NNPVLRPDEPGMN---VIEFRDPTTGWIGPD---GH
Q70LF5          QVTCLAEPADPSDPLLREWVKHPA-NPVVFPPPGIG----MKDFRDPTTAWFDAS--DGT
Q70XE6          QVQNLALPKNLSDPYLKEWIKLP-QNPLMAGTPTNNNNINASSFRDPSTAWQLSD---GK
Q7DLY6          QVQNLAYPEDPSDPLLLKWVKFSG-NPVLVPPPGIG----AKDFRDPTTAWKT---SSGK
Q7XA49          QVQNLAQPKNLSDPFLREWVKSP-KNPLMAPTSANK--INSSSFRDPTTAWLGKD---GH
Q7XAS5          QVQNLAYPTNLSDPLLLDWVKYKG-NPVLVPPPGIG----VKDFRDPTTAWTG--PQNGQ
Q7XZS5          QVQCLAVPADDADPLLTNWTKYEG-NPVLYPPPGIG----PKDFRDPTTAWFDPS--DNT
Q84LA1          QAQNIAFPKNRSDPYLREWIKAP-NNPVLRPDEPGMN---SIEFRDPTTGWIGPD---GL
Q84RM0          QVQCLATPADPSDPLLREWVKHPA-NPILYPPPGIG----LKDFRDPLTAWFDHS--DHT
Q84V21          QVQNYAIPANLSDPFLRKWIKPN-NNPLIVPDNSI----NRTEFRDPTTAWMGQD---GL
Q84XV1          QLQNVAYPKDLSDPYLREWVKPD-YNPVIAPGDGI----NASAFRDPTTAWRGPG---TH
Q8GT50          EVQNIAFPKNKSDPLLREWVKPR-GNPIIVPEGGI----NATQFRDPTTAWYADG----H
Q8GT63          QVQNVAYPKNASDPLLVDWVKPE-YNPVI-PVPADIK---RDDFRDPSTAWLGAD---GL
Q8GUA3          QVQNLAYPANLSDPLLIDWVKYRG-NPVMVPPPGIG----VKDFRDPTTAWTG--PQNGQ
Q8GUB8          QVQCLAVPEDPNDSLLRNWTKHEA-NPVLLPPPGIG----DKDFRDPTTAWFDES--DQT
Q8L6W0          QSQNLAFPKDPSDPLLREWVKSP-HNPVITAEDDIE----PSDFRDPTTAWQAVD---GT
Q8L6W1          QVQVLAYPKDTSDPNLKEWFLAP-QNPVMFPTPQNQ--INATSFRDPTTAWRLPD---GV
Q8L6W2          QVQNLAYPANLSDPLLLEWVKYPG-NPVLVPPPGIG----KLDFRDPTTAWLT---SEGK
Q8L897          QVQNLAYPADLNDPLLVDWIKYPS-NPVLVPPKGIL----PKDFRDPTTAWLT---TEGK
Q8LPM7          QVQNLAEPADPSDPLLIEWKKSNH-NPILMPPPGVG----PHDFRDPFPVWYNES--DSN
Q8LRN6          QVQNYAIPADLSDPFLRKWIKPD-NNPLIDADVNI----NKTQFRDPTTCWLGQD---GH
Q8RVH4          QVQNLAYPADPSDPLLIKWVKYPG-NPVLVPPPGID----NKDFRDPTTAWKT---PEGK
Q8VXS5          QVQVLAYPKDTSDPNLKEWFLAP-QNPVMFPTPQNQ--INATSFRDPTTAWRLPD---GV
Q8VXS7          QVQNLAYPANLSDPLLLEWVKYPG-NPVLVPPPGIG----KLDFRDPTTAWLT---SEGK
Q8W3M2          QVQNLAYPADPSDPLLIKWVKYPG-NPVLVPPPGIG----SKDFRDPTTAWLT---SEGK
Q93X59          QVQDLAWPKNLSDPFLREWVKHP-KNPLIIPPEGVK----DDCFRDPSTAWRGPD---GV
Q941I4          QVQNLAYPANLSDPLLLDWVKYSG-NPVLTPPAGIG----STDFRDPTTAWIG---PDGL
Q944C8          QVQNLAYPADPSDPLLLDWVKYPG-NPVLMPPRHIG----PKDFRDPTTAWAG---PDGK
Q944U7          QLQNYAVPANISDPHLENWIKPA-NNPLVAPDHTV----NRTAFRDPTTAWLGSD---GW
Q94C05          QVQNLAYPADLLDPLLLDWVKYSG-NPVLVPPPGIG----AQDFRDPTTAWKS--PNVGK
Q94C06          QVQNLAFPANLSDPLLVDWVKYPN-NPVIYPPPGIG----VKDFRDPTTAWTAG-SQNGQ
Q94C07          QDQNLADPVNITDPLLRDWVKTDV-NPVLYPPPGIG----AKDFRDPTTAFKENEVDDKR
Q94C08          QDQCLADPVNPTDPLLVDWVKYSV-NPVIYPPPGIG----VKDFRDPTTACRSPAGNDTR
Q9AUH1          EATCLALPADPNDPLLRRWTKHPA-NPIIWSPPGIG----TKDFRDPMTPWYDDS--DHT
Q9FNS9          QVQNLAFPKNLSDPLLKEWIKWS-GNPLLTPVDDIK----AGQFRDPSTAWMGPD---GK
Q9FQ62          QVQNLAYPEDLNDPLLLKWVKYSG-NPVLVPPPGIL----PKDFRDPTTAWKT---SDGK
```

```
Q9FR47           EVQCLAVPAEPNDPLLRRWTKHPA-NPVLVHPPGIK----DMDFRDPTTAWFDES--DST
Q9LD97           QVQNYAIPANLSDPFLRKWIKPN-NNPLIVPDNSI----NRTEFRDPTTAWMGQD---GL
Q9LDS8           QVQNYAIPANLSDPFLRKWIKPN-NNPLIVPDNSI----NRTEFRDPTTAWMGQD---GL
Q9M4K8           QVQNYAIPANLSDPFLRKWIKPN-NNPLIVPDNSI----NKTKFRDPTTAWMGQD---GL
Q9SBI2           QVQNVAYPKNVSDPLLREWVKPS-HNPVIVPEGGI----NATQFRDPTTAWRGPGP--EQ
Q9SM30           QLQCLAYAVNSSDPLLLEWKKYEG-NPILFPPPGVG----YKDFRDPSTLWRGP---DGD
Q9SPK0           QVQNVAYPKNVSDPLLREWVKPS-HNPVIVPEGGI----NATQFRDPTTAWRGPGP--EQ
Q9ZP42           QVQNLAFPKNLSDPFLREWVKVP-QNPLMAPTQANQ--INASSFRDPTTAWLGPD---KR
Q9ZR55           QVQNLAVPKDLSDPYLREWVKHT-GNPIISLPEEIQ----PDDFRDPTTTWLEED---GT
Q9ZR96           QLQCKAVPLNTSDPLLLEWVKYEN-NPILFTPPGIG----LKDYRDPSTVWTGP---DGK
Q9ZTW9           QDQNLAFPANLSDPLLVDWVKYPN-NPVIYPPPGIG----VKDFRDPTTAGTAG-MQNGQ
Q9ZTX2           QVQNLAFPANLSDPLLVDWVKYPN-NPVIYPPPGIG----VKDFRDPTTAWTAG-SQNGQ
P92916           QSINLAEAADPSDPLLLKWIKYDN-NPILWPPPGIV----RDDFRDPNPIWYNAS--EST
Q5FC15           QIMSVAMAADPSDPLLVEWVKYDEVNPVLRPPPGIG----LTDFRDPNPIWYNTT--DST
P93761           QVQNLAYPANLSDPLLLDWVKYQG-NPVLVPPPGIG----VKDFRDPTTAWTG--PQNGQ
P49175           QVQNLAEPADASDPLLREWVKSDA-NPVLVPPPGIG----PTDFRDPTTACRTPAGNDTA
Q43089           QVQNIAIPKNMSDPLLREWKKSP-KNPLMEPTIANK--INSSSFRDPTTSWLGKD---GF
Q39692           QVQNFAVPANYSDPYLREWIKPD-NNPIVR-ARSE----NSSSFRDPTTAWF-DG---VH
Q39693           QVQNYAVPANYSDPFLREWVKPD-NNPLVG-VHTEN----PSAFRDPTTAWF-DG---GH
Q56UD0           QVQNVAFPKNPRDPLLREWTKPG-YNPVI-PVPADVS---PDNFRDPTTAWLGSD---GL
Q0J360           QVQNVAFAKNPSDPLLREWEKPA-YNPVI-ALPADVP---GDKFRDPSTAWLGRD---GL
P29001           QVQNLAYPADPSDPLLLDWIKHTG-NPVLVPPPGIG----AKDFRDPTTAWLT---SEGK
O24509           QVQNLAYPADPSDPLLVDWIKHPG-NPVLVPPPGIG----AKDFRDPTTAWLT---SEGK
P29000           QVQNLAYPANLSDPLLLDWVKFKG-NPVLVPPPGIG----VKDFRDPTTAWTG--PQNGQ
Q43857           QVQNLAYPADLNDPLLVDWIKYPS-NPVLVPPPGIL----PKDFRDPTTAWLT---TEGK
P80065           QVQNLAYPANLSDPLLLDWIKYPD-NPVMFPPPGIG----STDFRDPTTAWIG--RDG-K


Conservation:    432347310111102222132316066124020001531100144449848567 6
SS                  IIIB          IIIC             IIID          IVA
TARGET           WRILIGSKK---RDRGIAYLYRS-LDFKKWFKAKHPLHS-VQGTGMWECPDFFPV
Q93X60           WRIVVGGDR---DNNGMAFLYQS-TDFVNWKRYDQPLSS-ADATGTWECPDFYPV
Q43866           WRVIIGSKI---HRRGLAITYTS-KDFLKWEKSPEPLHY-DDGSGMWECPDFFPV
A0A7Z0           WRITIGSKL---NKTGISLVYDT-KDFKTYEQLNGVLHA-VPGTGMWECVDFYPV
A3QRG0           WRTVIGSKDDD--GHAGIILSYKTKDFVNYELMPGNMHRGPDGTGMYECIDLYPV
A5GXL9           WRIVIGSEI--IKGQATALLYYS-TDGFNWTRSDKPLKF-SRETNMWECPDFYPV
A7IZK7           WRVIVGNK---LNRRGKALLYRS-KDFVRWTKAQHPLYS-IQGTGMWECPDFYPV
A7IZK8           WLVTLGSKV----NKTGIALVYETSDFKGYRLLDGVLHA-VPRTGMWECVDFYPV
A7LJR5           WYVVVGSKNDS-LSHTGIALVYTTKDFLSYTLLPGVLHAVDI-VGMWECVDLYPV
A7RDD3           WHVVIGSKDP---EHYGIVLIYTTKDFVNFTLLPNILHSTKQPVGMLECVDLFPV
A9CZQ1           WRLTVGTLIDEGG---LAIVYKS-KDFMKWVPAENPLYY-TNGSGMWECVDFFPL
A9E2W4           WRLTVGTVIDGNG---LAMLYKS-KDFVKWVPAENPLYY-TNGSGMWECVDFFPL
A9JIF3           WRIVVG--ADRD-NNGMAFLYQS-TDFVNWKRYEQPLSS-ADLTGTWECPDVYPV
A9LST6           WLLTIGSKIG----KTGVALVYETSNFTSFKLLDGVLHA-VPGTGMWECVDFYPV
A9YTS8           WRMVMGSKHNQT---IGCALVYRTTNFTHFVLNEEVLHA-VPHTGMWECVDLYPV
A9YTS9           HRMIMGTKINR----TGLVLVYHTTDFVNYVMLDEPLHS-VPNTDMWECVDFYPV
B0I1Q7           WRIIIGSKDDD--GHAGIILSYKTTDFVNYELMPGTMHRGPDGTGMYECLDIFPV
B2NIA0           WRITIGSKF----NTTGISMVYTTNDFINYELHNGVLHE-VPGTGMWECVDFYPV
O04372           WRIAIGTKDA---DHSGVALVYSTKDFLNYTLLPGTLHTVKH-VGMWECIDFYPI
O23786           YRMVMGSKHNET---IGCALIYHTTNFTHFELKEEVLHA-VPHTGMWECVDLYPV
O24459           WRMVMGSKHNET---IGCALVYRTTNFTHFELNEEVLHA-VPHTGMWECVDLYPV
O65341           WRIVIGSKDDAEGDHAGIAVVYRTRDFVHFELLPDLLHR-VAGTGMWECIDFYPV
O65342           WRIVIGSKDDAEGDHAGIAVVYRTRDFVHFELLPDLLHR-VAGTGMWECIDFYPV
O65778           HRMIIGTKR----NTTGLVLVYHTTDFTNYVMLDEPLHS-VPNTDMWECVDLYPV
O81082           WHMLIGSKDD---NHYGTVLIYTTKDFETYTLLPDILHKTKDSVGMLECVDLYPV
O81083           WRIVIGSKDS---SHSGIAIVYSTKDFINYKLIPGILHAVER-VGMWECVDFYPV
O81118           WRLVVGSK---ENMRGIAVLYRS-RDFRRWIKAHHSLHA-G-LTGMWECPDFYPV
O81985           HRMIMGTKRGN----TGMVLVYYTTDYTNYELLDEPLHS-VPNTDMWECVDFYPV
O81986           YRMVMGSKHNET---IGCALIYHTTNFTHFELKEEVLHA-VPHTGMWECVDLYPV
O82119           WRVLVGSVR---NHRGKVIMYKSNKNFMKWTKAKHPLHS-AQGTGNWECPDFFPV
Q05G13           WRTIIGSKDDD--GHAGIVLSYKTTDFVNYELMPGNMHRGPDGTGMYECLDIYPV
Q05JI1           WRLTIGSKIG----KTGISLVYQTTDFKTYELLDEYLHA-VPGTGMWECVDFYPV
Q05JI2           WRIAIGSRI----NRTGITFVYDTKDFINYELLRGVLHG-VPNTGMWECVDFYPV
Q0PCC5           HRMIMGTKVNR----TGLVFVYHTTDFINYVLLDEPLHS-VPNTDMWECVDFYPV
Q0PCC7           HRMIMGTKRGS----TGMVLVYYTKDFTKYELKDEPLHS-VANTDMWECVDFYPV
Q0PCC8           HRMIMGSKRNN----TGLVLVYYTTDFTNYELLDEPLHS-VPNTDMWECVDFYPV
Q0PCC9           HRMIIGTRRNN----TGLVLVYHTKDFINYELLDEPLHS-VPDSGMWECVDLYPV
Q0W9N0           WRITIGSKI----NKTGISLVYDTIDFKKFELLKGMLHG-VPGTGMWECVDFYPV
Q1KL65           WLLTIGSKIG----KTGIALVYETSNFTSFKLLDEVLHA-VPGTGMWECVDFYPV
Q2UXF7           WRIAVAAKVGGPNGIASTLIYRS-KDFRHWKRNASPLYT-SRAAGMVECPDLFPV
Q2WEC6           HRMIMGSKRGN----TGVVLVYHTTDFTNYELLDEPLHS-VPNTNMWECVDFYPV
```

```
Q2XQ19          WRVVIGSKD---AHHAGIAMTYKTKDFVHYELVPGLLHR-VPATGMWECIDFYPV
Q2XQ21          WRLLVGSVVTGGS-RGVAYVYRS-RDFRLWTRVRRPLHS-A-PTGMWECPDFYPV
Q3L7K5          WRVIIGSK---IKRKGLAILYRS-KDFVRWTKAQHPLHS-GKNTGMWECPDFFPV
Q41215          WLLTIGSKIG----KTGVALVYETSNFTSFKLLDGVLHA-VPGTGMWECVDFYPV
Q41604          WKLAIGSKDE---QHNGISLIYRTYDFVSYELLPILLHA-VEGTGMWECVDFYPV
Q41606          WKLAIGSKDE---QHNGISLIYRTYDFVSYELLPILLHA-VPGTGMWECVDFYPV
Q42691          WRVLIGKSK--RRQRGLSLLYRS-RDFVHWVKAKHPLYS-YERSGMWECPDFFPV
Q42722          WRLIIGSKL----NKTGISLVYDTVDFKNFTLLDGVLHA-VHGTGMWECVDFYPV
Q43172          WRTLIGSV---WGKQGLAILYKS-KNFMKWTKIQHPLHS-VDGTGNWECPDFFPV
Q43799          WRILVGSV---KNHRGKVILYRS-KDFMKWTKAKHPLHT-ATNTGNWECPDFFPV
Q43818          SLNITGSDLG----RGGSAVLYSETEFIKHGKTGVPIDA-QTNTVTSEKLDFVLV
Q43855          WRILIGGKT---GDTGVAYLYRS-KNFLKWIRAKHPIHS-AKRTGMWECPDFYPV
Q43856          WRGIVGSKR---STRGIAILYKS-KDFVKWKKSKHPLHS-AKGTGMWECPDFFPV
Q4AEI9          WRIIIGSKNDT--DHSGIVFTYKTKDFVSYEMIPGYLYRGPAGTGMYECIDLYAV
Q547Q0          WLLTIGSKIG----KTGVALVYETSNFTSFKLLDGVLHA-VPGTGMWECVDFYPV
Q575T1          WRLVIGSKD---DHHAGMVMTYKTKDFIDYELVPGLLHR-VPGTGMWECIDLYPV
Q5ZQK6          WRVLVGSK--KN-DLGVAYLYQS-KDFVKWERFDYPLMS-MMETSTWECPDFFPV
Q64GB3          WRIAIGAEVNGYS---AALLYKS-EDFMNWSRVGHPLYS-SSASTMWECLDFFAV
Q6F4N3          WRTIIGSKDDD--GHAGIVLSYKTTDFVNYELMPGNMHRGPDGTGMYECLDIFPV
Q6KCH6          WRLVIGPKD---DRHAGMVMTYKTKDFMDYELVPGLLHR-VPGTGMWECIDLYPV
Q6PVN1          WRTVIGSKDN--HGHTGIVMTYKTKDFINYELIPGLLHS-VPGTGMWECIDFYPV
Q70AT7          WRMAVGGELNGYS---AALLYKS-EDFLNWTKVDHPPYS-HNGSNMWECPDFFAA
Q70LF5          WRTIIGSKNDS--DHSGIVFSYKTKDFVSYELMPGYMYRGPKGTGEYECIDLYAV
Q70XE6          WRVIVG---TQQGKRGLAVLFTS-DDFVKWNNTGNPLHS-TEGNGIWECPDFFPV
Q7DLY6          WRITIGSKI----NRTGISLIYDTTDFKTYEKHETLLHQ-VPNTGMWECVDFYPV
Q7XA49          WRVLVGSKR---RTRGIAILYRS-KDFVNWVQAKHPLYS-ILGSGMWECPDFFPV
Q7XAS5          WLLTIGSKIG----KTGIALVYETSNFTSFKLLGEVLHA-VPGTGMWECVDFYPV
Q7XZS5          WRIVIGSKDDAEGDHAGIAVVYRTKDFVHFELLPDLLHR-VAGTGMWECIDFYPV
Q84LA1          WRMAVGGELNGYS---AALLYKS-EDFLNWTKVDHPLYS-HNGSNMWECPDFFAV
Q84RM0          WRTVIGSKDDD--GHAGIILSYKTKDFVNYELMPGNMHRGPDGTGMYECIDLYPV
Q84V21          WRILIA---SMRKHRGMALLYRS-RDFMKWIKAQHPLHS-STNTGNWECPDFFPV
Q84XV1          WKLVVG---SKWNRRGKAILYRS-RDFVHWVKAKHPLHT-VKDTGMWECPDFYPV
Q8GT50          WRLLIG-ALSGAS-RGVAYVYRS-RDFMRWTRVRKPLHS-A-PTGMWECPDLYPV
Q8GT63          WRIAVAARVHDVG--GATLIYRS-KDFLRWERNADPLYL-AHAAGMVECPDLFPV
Q8GUA3          WLLTIGSKIG----KTGIAIVYGTSNFTNFKLLDGVLHA-VPGTGMWECVDFYPV
Q8GUB8          WRTVIGSKDN--NGHAGIAMVYKTKDFLNYELIPGYLHR-VDGTGMWECIDFYPV
Q8L6W0          WQVLIG--GKID-GRGMAYLYQS-NDFINWTRSEKIFHS-SVKTGMWECPDFYPV
Q8L6W1          WRLLIG---SKRGQRGLSLLFRS-RDFVHWVQAKHPLYS-DKLSGMWECPDFFPV
Q8L6W2          WRITIGSKI----NKTGISLVYDTTDFKNYELLSNILHA-VQGTGMWECVDFYPV
Q8L897          WRITIGSKI----NKTGVALVYDTVDFKTYERKDVLLNA-VPGTGMWECVDFFPV
Q8LPM7          WHMLIGSKDD---NHYGTVLIYLTKDFKTYTLLPEILHKTKDNVGMLECVDLYPV
Q8LRN6          WRTLIGSLW---GNKGMAILYKS-RDLMKWTKVQQPLHS-VDGTGNWECPDFFPV
Q8RVH4          WRITIGSKI----NKTGISLVYDTEDFKTFELLDGLLHA-VPGTGMWECVDFYPI
Q8VXS5          WRLLIGSKR---GQRGLSLLFRS-RDFVHWVQAKHPLYS-DKLSGMWECPDFFPV
Q8VXS7          WRITIGSKL----NKTGISLVYDTTDFKNYELLSNILHA-VQGTGMWECVDFYPV
Q8W3M2          WRIAIGSRI----NRTGITFVYDTKDFINYELLRGVLHG-VPNTGMWECVDFYPV
Q93X59          WRIVVG--GDRD-NNGMSLLYQS-TDFVNWKRYDQPLSS-AIATGTWECPDFYPV
Q941I4          WRTTIGSKV----NKTGISIVYTTTNFIDYELLEGVLHA-VPGTGMWECVDFYPV
Q944C8          WRLTIGSKIG----KTGISLVYQTTDFKTYELLDEYLHA-VPGTGMWECVDFYPV
Q944U7          WRMLVGSK---NKRRGIAHLYKS-KDFMNWVKAKHPIHS-RPDTGMWECPDFFPV
Q94C05          WLLTIGSKV----NKTGISLVYETTNFTDYKLLDGVLHA-VPGTGMWECVDFYPV
Q94C06          WLVTIGSKVG----KTGISLVYETTNFTTFKLLDGVLHA-VPGTGMWECVDLYPV
Q94C07          WRAIIGSKEKE---KVGLSVVYKTDNFSHFRPVPVIMHR-VPGTGMWECVDFYPV
Q94C08          L-ITIGSKLG----KTGISLVYETTNFTTFKLLYGVLHA-VPGTGMWECVDLYPV
Q9AUH1          WRTLFGSKDDHHGHHDGIAIMYKTKDFLNYELIPGILHR-VENTGEWECIDFYPV
Q9FNS9          WRIVIG--SEID-GHGTALLYRS-TNGTKWIRSKKPLHF-SSKTGMWECPDFYPV
Q9FQ62          WRITIGSKI----NKTGISLVYDTIDFKTYEKHDTLLHK-VPNTGMWECVDFYPV
Q9FR47          YRTVIGSKDDNQGDHAGFAMVYKTKDFLSFQRIPGILHR-VEHTGMWECMDFYPV
Q9LD97          WRILIASMR---KHRGMALLYRS-RDFMKWIKAQHPLHS-STNTGNWECPDFFPV
Q9LDS8          WRILIGSMR---KHRGMALLYRS-RDFMKWIKAQHPLHS-STNTGNWECPDFFPV
Q9M4K8          WRIVIGSMR---KHRGMALLYRS-RDFIKWAKAQHPLHS-SPHTGNWECPDFFPV
Q9SBI2          WRLLVGSAAGSMPPRGVAYVYRS-RDFRRWRRVRRPLHS-A-PTGMWECPDFYPV
Q9SM30          WIMIMGSKH---NQTIGCALVYRTSNFTHFELSEEPLHA-VPHTGMWECVDLYPV
Q9SPK0          WRLLVGSAAGSSP-RGVAYVYRS-RDFRRWRRVRRPLHS-A-PTGMWECPDFYPV
Q9ZP42          WRLIIGSK---RNHRGLAILYRS-KDFMHWTKAKHPLYS-TPKNGMWECPDFFPV
Q9ZR55          WRLLVGSQK--D-KTGIAFLYHS-GDFVNWTKSDSPLHK-VSGTGMWECVDFFPV
Q9ZR96          HRMIMGTKINR----TGLVLVYHTTDFTNYVMLEEPLHS-VPDTDMWECVDLYPV
Q9ZTW9          RLVTIGSKVG----KTGISLVYETTNFTTFKLLYGVLHA-VPGTGMWECVDLYPV
Q9ZTX2          WLVTIGSKVG----KTGISLVYETTNFTTFKLLDGVLHA-VPGTGMWECVDLYPV
P92916          YHIVVGSKNDS-LQHTGIALVYLTKDFKKFDLLPTVLHSVDK-VGMWECVEVYPV
Q5FC15          WQLVIGSKNDS-LQHTGIAMVYTTKDFINLTLLPGVLHSVDH-VGMWECVDLFPV
```

```
P93761          WLLTIGSKVG----KTGIALVYETSN---FKLLDGVLHA-VPGTGMWECVDFYPV
P49175          WRVAIGSKDR---DHAGLALVYRTEDFVRYDPAPALMHA-VPGTGMWECVDFYPV
Q43089          WRVLIGSKI---DTKGMAILYKS-KNFVDWVEAKHPLHS-AEGTGMWECPDFYPV
Q39692          WKILVGSRR---KHRGIAYLYRS-RNFLKWTKAKHPLHS-KDRTGMWECLDFYPV
Q39693          WKMLVGSSR---KHRGIAYLYRS-KDFKKWKRSPHPIHT-KAETGMWECPDFYPV
Q56UD0          WRFAISAVADGVG---ATLVYRS-ADFLRWERNAAPLHA-SRDAVMAECPDLFPV
Q0J360          WRIAVSAEVDGVA---STLVYRS-KDFVRWERNAAPLHA-SRAAGMVECPDLFPV
P29001          WRITIGSKL----NKTGIALVYDTEDFKTYELKEGLLRA-VPGTGMWECVDFFPV
O24509          WRITIGSKL----NKTGIALVYDTDDFKTYELKNGHLRA-VPGTGMWECVDFFPV
P29000          WLLTIGSKIG----KTGVALVYETSNFTSFKLLDGVLHA-VPGTGMWECVDFYPV
Q43857          WRITIGSKI----NKTGVALVYDTVDFKTYERKDMLLNA-VPGTGMWECVDFFPV
P80065          WRITIGSKV----NKTGISLMYKTTDFITYELLDNLLHA-VPGTGMWECVDFYPV


Conservation:   2002000000000222210011012123455592722212162634815110131513 12
SS                                             IVB          IVC        IVD
TARGET          SLSGEE------GLDTSVG-GSN----VRHVLKVSLDLTRYEYYTIGTYDEKKDRYYPDE
Q93X60          PLNSTN------GLDTS-VYGGS----VRHVMKAGFEG--HDWYTIGTYSPDRENFLPQN
Q43866          TRFGSNG------VETSSFGEPNEI--LKHVLKISLDDTKHDYYTIGTYDRVKDKFVPDN
A0A7Z0          SKTS---------DKGLDT--SVNGPDVKHVVKASLDDDRNDYYSLGSYEEKTGKWVPDN
A3QRG0          GGNS------------SEMLGGDDSPGVLFVLKESSDDERHDYYALGRFDAVANVWTPID
A5GXL9          SNTGKD------GIDTS-FQGNN----TMHVLKVSFDS--HDYYVIGMYDPQMDQFLLAT
A7IZK7          SS-SPIG------LDTSTIGEG-----VKHVLKVSLDDTKHDQYAIGTYVHSKDVFVPNA
A7IZK8          STTG---------DNGLDT--SANGPGTKHVLKASLDENKHDYYALGTYDPKNNKWTPDD
A7LJR5          ATAG---PLVGRALEN----SVPAGENVKHVLKAGLNDEWHDYYAIGTYDREANKWTPDD
A7RDD3          ATTD---SRANQALD---MTTMRPGPGLKYVLKASMDDERHDYYALGSFDLDSFTFTPDD
A9CZQ1          K-----------------EIQGA----TKYLLKVSMYDTLHDYYVMGTYDEERDIF-IKD
A9E2W4          G-----------------EARGV----TKYMLKVSMFDVSYDYYALGTYDEEGGVF-TRD
A9JIF3          PLNSTN------GLDTS-VYGGX----VKHVMKAGFGG--HDWYTIGTYTPDRENFLPQN
A9LST6          STKK---------TNGLDT--SYNGPGVKHVLKASLDDNKQDHYAIGTYDLGKNKWTPDN
A9YTS8          STTH---------TNGLEMK--DNGPDVKYILKQSGDEDRHDWYAIGSFDPINDKWYPDD
A9YTS9          STINDS---------ALDIA--AYGSDIKHVIKESWEGHGMDWYSIGTYDAMKDKWTPDN
B0I1Q7          GGNS------------SEMLGGDSSPEVLFVLKESANBEWHDYYAPWEVRCRRNTWTPQD
B2NIA0          AING---------SKGLDT--SVNSGRIKHVLKASLDDTKLDHYAIGTYFIENETWVPDD
O04372          ATSG---AGANRGLDP----SVRPSKLVKHVLKESSDDDRQDWYAIGTYDPDTNKWTPDD
O23786          STTH---------TNGLDMV--DNGPNVKHVLKQSGDEDRHDWYALGTYDVVNDKWYPDD
O24459          STTH---------TNGLDMK--DNGPNVKYILKQSGDEDRHDWYAVGTFDPEKDKWYPDD
O65341          ATRGKVSGNGVDMSDALGKNG-AVVGDVVHVMKAGMDDDRHDYCALGRYDAAANAWTPLD
O65342          ATRGKVSGNGVDMSDALGKNG-AVVGDVVHVMKAGMDDDRHDYCALGRYDAAANAWTPLD
O65778          STTNDS---------ALDVA--AYGPGIKHVLKESWEGHAMDFYSIGTYDAFNDKWTPDN
O81082          ATTG---NQIGNGLE---MKGG-SGKGIKHVLKASMDDERHDYYAIGTFDLESFSWVPDD
O81083          ATADS--SHANHGLDP----SARPSPAVKHVLKASMDDDRHDYYAIGTYDPAQNTWVPDD
O81118          AVAGGRRHH-RSGVDTRELHDSTVAAEVKYVLKVSLDVTRYEYYTIGWYDHAKDRYTPDL
O81985          SLTNDS---------ALDMA--AYGSGIKHVIKESWEGHGMDWYSIGTYDAINDKWTPDN
O81986          STVH---------TNGLDMV--DNGPNVKYVLKQSGDEDRHDWYAIGSYDIVNDKWYPDD
O82119          SLKNEN------GLDTSYD-GKD----VKHVLKVSFDVTRFDHYTVGTYDTKKDKYFPDN
Q05G13          GGNS------------SEMLGGDSSPEVLFVLKESANDEWHDYYALGWFDAAANTWTPQD
Q05JI1          AING---------SVGLDT--SATGPGIKHVLKASLDDTKVDHYAIGTYNPANDKWTPDN
Q05JI2          STTG---------EHGLDT--SHNGPGVKHVVKASMDDDRHDYYAIGTYHEKNVTWVPDN
Q0PCC5          STID---------DSALDMA--AYGSDIKHVIKESWEGHGMDWYSIGTYDAMKDKWTPDN
Q0PCC7          SLTNDS---------ALDMA--AYGSGIKHVIKESWEGHGMDWYSIGTYDAKTDKWTPDN
Q0PCC8          SLTNDS---------ALDMA--AFGSGIKHVIKESWEGHGKDFYSIGTYDPKIDKWIPDN
Q0PCC9          STMNDT---------ALDVA--AYGSGIKHVLKESWEGHAKDFYSIGTYDAINDKWWPDN
Q0W9N0          SKIA---------ENGLDT--SENGPAVKHVLKSSLDDDRNDYYALGTYNAGAGKWVPDN
Q1KL65          STEK---------TNGLDT--SYNGPGVKHVLKASLDDNKQDHYAIGTYDLTKNKWTPDN
Q2UXF7          AEPGVEEGR-LGYASGPASGA------VRHVLKLSVMNTTQDYYAVGRYDDVADTF-VPE
Q2WEC6          SLTNDS---------ALDIA--AYGPGIKHVIKESWEGHAMDFYSIGTYDAITDKWTPDN
Q2XQ19          GTRG---DNGIDMSEAMAKS--NNAEDVVHVMKASMDDDRHDYYALGRYDAAANTWAPMD
Q2XQ21          TADGR-----QHGLDTSVMANDKRP--VKHVLKNSLDLRRYDYYTVGTYDREAERFVPDD
Q3L7K5          SINSSTG------VDTSSISKT-----LKYVLKLSLDDTKHDYYTIGSYNREKDTYVPDK
Q41215          STKK---------TNGLET--SYNGPGVKHVLKASLDDNKQDHYAIGTYDLGKNKWTPDN
Q41604          LTNS----------TVGLDTSVPPGPGVRHVLKASLDDDKHDYYAIGTYDVVSGTWIPDD
Q41606          LTNS----------TVGLDTSVPPGPGVRHVLKASLDDDKHDYYAIGTYDVVSGTWTPDD
Q42691          YKNGNTMG-----IDTSVIGPN-----IKHVLKVSLDVSKHDVYTIGGYDTKKDAYTPDV
Q42722          SKFG---------ENGLDT--SFDGVGVKHVMKASLDDDRNDYYAIGTYDPVSGKWVPDN
Q43172          LLHGTN------GLDASYN-KKN----IKHALKVSLDVTRFEYYTVGIYDTKKDRYIPDK
Q43799          SLKHTN------GLDTSYR-GEY----TKHVLKVSLDVTRFEYYTVGTYDTRKDRYIPDN
Q43818          HAPG-----------ITGMWTSVECRSVVGIAKNSLDTSQYNGDLVGSILTSWDKNHETN
Q43855          SLEGKN------GLDLSMMMGNN----VKHVLKNSLDITRYEYYTIGTYLQNQDKYIPDK
Q43856          LKNGIK--------GVDTSLN-DDY--VRHVLKVSLDDKKHDYYLIGSYDEEKDRFVPDR
Q4AEI9          GGGRK----------ASDMYNST-AKDVLYVLKESSDDDRRDYYALGRFDAAANTWTPID
```

```
Q547Q0          STKK---------TNGLDT--SYNGPGVKHVLKASLDDNKQDHYAIGTYDLGKNKWTPDN
Q575T1          GGLR-----GIDMTEAVAAASNNGGGDVLHVMKESSDDDRHDYYALGRYDAAKNTWTPLD
Q5ZQK6          SVSSTN------GLDTSGVINPG----VKHVVKVGFNG--IDWYTIGTLS-ERDNYVPEN
Q64GB3          LPGSNGGLD-L-----SAAIPKG----AKHVLKFSVDQ--CDKYMIGVYDLECDAF-VPD
Q6F4N3          GGNS------------SEMLGGDSSPEVLFVLKESANDEWHDYYALGWFDAAANTWTPQD
Q6KCH6          GGVR-----GIDMTDAVTAASNNGGDDVLHVMKESSDDDRHDYYALGRYDATKNTWTPLD
Q6PVN1          GGAD-------------------GSEELYVMKESSDDDRHDWYALGRYDAAANKYTPID
Q70AT7          LPGNNGGLD-L-----SAAIPQG----AKHALKMSVDSV--DKYMIGVYDLQRDAF-VPD
Q70LF5          GGGSR----------ASDMYNST-AEDVLYVLKESSDDDRHDWYSLGRFDAAANKWTPID
Q70XE6          Y-VGKSLG-----ADTSIIGDD-----VKHVLKLSLFDTQYEYYTIGRYDIEKDIYVPDE
Q7DLY6          SKTQ---------LNGLDT--SVNGPDVKHVIKASMDDTRIDHYAIGTYDDSNATWVPDN
Q7XA49          LNNDQL--------GVDTSVN-GYD--VRHVLKVSLDDKKHDYYMIGSYNAAKDAFIPD-
Q7XAS5          STEK---------TNGLDT--SYNGPGVKHVLKASLDDNKQDHYAIGTYDLTKNKWTPDN
Q7XZS5          ATRGKASGNGVDMSDALGKNG-AVVGDVVHVMKASMDDDRHDYYALGRYDAAANAWTPLD
Q84LA1          LPGNNAGLD-L-----SAAIPQG----AKHALKMSVDSV--DKYMIGVYDLQRDAF-VPD
Q84RM0          GGNS------------SEMLGGDDSPGVLFVLKESSDDERHDYYALGRFDAVANVWTPID
Q84V21          LFNSTN------GLDVSYR-GKN----VKYVLKNSLDVARFDYYTIGMYHTKIDRYIPNN
Q84XV1          AVKG------RRGLDTSAYGDG-----VKHVLKVSLDLRKYEYYTLGKYYHYQDKYVPDN
Q8GT50          TADGRHR---HKGLDTSVVSGPR----VKHVLKNSLDLRRYDYYTVGTYDRKTERYVPDN
Q8GT63          SEPGVEVG-------LPASGAG-----ARHVLKMSVMDTVQDYYVVGRYDDAADTF-VPE
Q8GUA3          STDE---------ANGLDT--SYNGPGIKHVLKASLDDDKHDYYAIGTYDPVKNKWTPDN
Q8GUB8          GGKNGS--------------------EELYVIKESSDDDRHDWYTLGKYDAAANTFTAAD
Q8L6W0          SINGKD------GVDNY-LEKGN----TKFVLKASFLD--HDHYILGYYKAERNGFQVEA
Q8L6W1          YANGDQMG-----VDTSIIGSH-----VKHVLKNSLDITKHDIYTIGDYNIKKDAYTPDI
Q8L6W2          SVAE---------PNGLDT--STNDQSVKHVLKASMDDDRNDYYTLGTYIEDNVTWVPDN
Q8L897          SKKS---------ENGSDT--SINGVEVKHVMKVSLDDDRHDYYSLGTYDEKKVKFIADD
Q8LPM7          ATTG---NQIGNGLE---MQVG-FGKGIKHVLKASMDDERHDYYAIGTFDLESFTWVPDD
Q8LRN6          LLRGTN------GLDASYK-GEN----IKYVLKVSLDVTRFEYYTVGIYDTKKDKYIPDK
Q8RVH4          SKQG---------ENGLDT--SVDGPGVKHVVKASMDDDRNDYYAIGTYDAYKGKWTPDN
Q8VXS5          YANGDQMG-----VDTSIIGSH-----VKHVLKNSLDIPKHDIYTIGDYNIKKDAYPPDI
Q8VXS7          SVAE---------PNGLDT--STNGPSVKHVLKASMDDDRNDYYTLGTYIEDNVTWVPDN
Q8W3M2          STTG---------EHGLDT--SHNGLGVKHVVKASMDDDRHDYYAIGTYHEKNVTWVPDN
Q93X59          PLNSTN------GLDTS-VYGGS----VRHVMKAGFEG--HDWYTIGTYSPDRENFLPQN
Q941I4          SING---------STGLET--SVNGPGVKHVLKASLDDTKMDHYAIGTYFLENNTWIPDD
Q944C8          AING---------SVGLDT--SATGPGIKHVLKASLDDTKVDHNKTGTYNPENDKWTPDN
Q944U7          PKSGEN------GLDVGIT-GRD----VRHVLKVSLDLTRYEYYTIGRYYPEIDRYIPYD
Q94C05          STIG---------AIGLDT--SANVPGIKHVLKASLDDDKHDYYAIGTYDPFNNTWTPDN
Q94C06          STTG---------ENGLDT--SVNGLDVKHVLKASLDDDKHDYYALGTYDPAKNKWTPDN
Q94C07          STVADV--ATDEGSDSTEY--SVPGIGVKHVLKSRLDDDKDDYKALGTYFAATGTFAADD
Q94C08          STKSVI--VITQ-ENGLDT--SVHGLGVKHVLKTSFDDDKHDYYALGTYDLQADTFVPDD
Q9AUH1          GGGGSENSS-----------------EVLHVLKASMDDERHDYYSLGTYDSAANIWTPID
Q9FNS9          TNGDKK------GLDTS-VQGNN----TLHVLKVSFNSR--EYYVIGTYDPIKDKFSVVT
Q9FQ62          SKTA---------VNGLDT--SVNGPNVKHIVKASMDDTRFDHYAVGTYFDSNGTWIPDD
Q9FR47          GGGDNSS------------------SEVLYVIKASMNDERHDYYALGRYDAAGNTWTPLD
Q9LD97          LFNSTN------GLDVSYR-GKN----VKYVLKNSLDVARFDYYTIGMYHTKIDRYIPNN
Q9LDS8          SLNSTN------GLDVSYR-GKN----VKYVLKNSLDVARFDYYTIGMYHTKIDRYIPNN
Q9M4K8          SLKNTN------GLDASYR-GKN----VKYVLKNSLDVNRFEYYTIGMYDTKKDRYIPDN
Q9SBI2          SKGGAP----RAGLETSVPPGPR----VKHVLKNSLDLRRYDYYTVGTYHPRAERYVPDD
Q9SM30          STTH---------TNGLDMM--DNGPNVKYILKQSGDEDRHDWYAIGSFDPINDKWYPDD
Q9SPK0          SKGGAP----RAGLETSVPPGPR----VKHVLKNSLDLRRYDYYTVGTYHPRAERYVPDD
Q9ZP42          SKTKLLG------LDTSAIGPD-----VKHVLKVSLDNTRKEYYTIGTYNVSKDIYIPDD
Q9ZR55          WVDSTN------GVDTS-IINPSNR--VKHVLKLGIQDHGKDCYLIGKYSADKENYVPED
Q9ZR96          STINDS--------ALDIA---AYGPDMKHVIKESWEGHGMDWYSIGTYDVINDKWTPDN
Q9ZTW9          STTG---------ENGLDT--SVNGLGVKHVLKTSLDDDKHDYYALGTYDPVKNKWTPDN
Q9ZTX2          STTG---------ENGLDT--SVNGLDVKHVLKASLDDDKHDYYALGTYDPAKNKWTPDN
P92916          ATTG---PLLHKAIDNFDVDRVLDRSTVKHVLKASMNDEWHDYYAIGTFDPIGNKWTPDD
Q5FC15          ASSG---PLIGRGLD----RSMMLADNVKHVLKASMNDEWHDYYAIGSYDVATHRWVPDD
P93761          STLD---------ANGLDT--SYNGPGIKHVLKASLDDNKQDHYVIGTYDPVKNKFSPDN
P49175          AAGSG----AAAGSGDGLETSAAPGPGVKHVLKASLDDDKHDYYAIGTYDPATDTWTPDS
Q43089          LDKNLLR------TGVDTSRNGDDD--VRHVLKVSLDDTKHDHYLIGSYDVVKDVFVPEN
Q39692          APKGMN------GLDTSVT-GQD----IKHVLKVSLYSTRYEYYTVGEYDRDNDIYVPDN
Q39693          SPRSED------GLDNSKM-GRG----IKHVLKVSLNSTRYEYYTIGRYNRVRDFYVPDN
Q56UD0          AEHGEDGLD-LDASAIGGAGAG-----VRHVLKVSMPDTLEDYYMVGRYDDADDTFTVPP
Q0J360          AERGEDGLD------TSANGAGG----VRHVLKLSVMDTLQDYYMVGTYDDAADAF-SPA
P29001          SKKN---------GNGLDT--SVNGAEVKHVMKVSLDDDRHDYYAIGTYDDNKVLFTPDD
O24509          SKKN---------ENGLDTSLSINGAEVKYVMKVSLDDDRHDYYTIGTYDENKVLFTPDD
P29000          STKK---------TNGLDT--SYNGPGVKHVLKASLDDNKQDHYAIGTYDLGKNKWTPDN
Q43857          SMKS---------ENGLDT--SFTGDEVKHVMKVSLDDDRHDYYALGTYDEKKVKFIADD
P80065          SVTG---------SNGLDT--SVNGPGVKHVLKSSLDDDRHDYYALGTYDPINDKWTPDN
```

```
Conservation:   011110203011125274912369776756103167544543274330014110578534
SS                                        VA            VB                  VC
TARGET          A-----LVDGWAGLRYDYG-NFYASKTFFDPSKNRRILWGWANESDSVQQD-MNKGWAGI
Q93X60          GL---SLTGSTLDLRYDYG-QFYASKSFFDDAKNRRVLWAWVPETDSQADD-IEKGWAGL
Q43866          G-----FKMDGTAPRYDYG-KYYASKTFFDSAKNRRILWGWTNESSSVEDD-VEKGWSGI
A0A7Z0          QK---IDVGIG--IRYDYG-IFYASKTFYDQNKERRVLWGWIGESDSENAD-MQKGWASV
A3QRG0          RE---LDLGIG--LRYDWG-KYYASKSFYDQKKNRRIVWAYIGETDSEQAD-ITKGWANL
A5GXL9          S----DFNVSNTQLQYDYG-RFYASKSFYDGAKKRRVLWGWVNEGDNPSDA-FKKGWSGL
A7IZK7          G-----AAEKFSGLRYDYG-KSYASKTFYDSLKKRRILWGWINESLSREDY-IAQGWSGV
A7IZK8          PE---LDVGIG--LRLDYG-KYYASKTFYDQNKKRRILWGWIGETDSEAAD-LMKGWASV
A7LJR5          EI---IDVGIG--LRYDWG-KFYASRTFYDPVKQRRVLWGYVGETDSREVD-IRKGWASV
A7RDD3          ET---IDVGVG--LRYDWG-KFYASKTFYDQEKHRRVLWGYVGEVDSKRDD-ALKGWASL
A9CZQ1          DASSD---DCRMWPMIDYG-RLYASKTFVDEAKQRRILWAWSNETSSVADN-VAKGWAGI
A9E2W4          DASST---DYRTWPMIDYG-RVYASKTFLDEAKQRRILWGWSNESNSIADD-VAKGWAGI
A9JIF3          EV---DRKYYGPEVR--YG-NFYASKSFFDDAKNRRVLWGWIPESDSQEDD-IQKGWAGL
A9LST6          PE---LDCGIG--LRLDYG-KYYASKTFYDPKKERRVLWGWIGETDSESAD-LQKGWASV
A9YTS8          PE---NDVGIG--LRYDYG-KFYASKTFYDQHKKRRVLWGYVGETDPPKDD-LLKGWANI
A9YTS9          PE---LDVGIG--LRVDYG-RFFASKSLYDPLKKRRVTWGYVAESDSADQD-LNRGWATI
B0I1Q7          PE---ADLGIG--LRYDWX-KYYASKSFYDPTKNRRIVWAYVGETDSEQAD-KAKGWASL
B2NIA0          PT---IDVGIG--LRYDYG-RYYASKTFYDQNKDRRILLGWVNETDTETDD-LKKHWASL
O04372          ES---LDVGIG--LRYDLG-KFYASKTFYDQEKKRRVLWGWIGESDSESAD-ILKGWASL
O23786          PE---NDVGIG--LRYDFG-KFYASKTFYDQHKKRRVLWGYVGETDPPKYD-VYKGWANI
O24459          PE---NDVGIG--LRYDYG-KFYASKTFYDQHQKRRVLWGYVGETDPPKSD-LLKGWANI
O65341          AE---KDVGTG--LRYDWG-KFYASKTFYDPAKRRRVLWGWVGETDSERAD-VSKGWASL
O65342          AE---KDVGTG--LRYDWG-KFYASKTFYDPAKRRRVLWGWVGETDSERAD-VSKGWASL
O65778          PE---LDVGIG--LRCDYG-RFFASKSLYDPLKKRRVTWGYVAESDSYDQD-VSRGWATI
O81082          DT---IDVGVG--LRYDYG-KFYASKTFYDQEKKRRILWGYVGEVDSKADD-ILKGWASV
O81083          AS---VDVGIG--LRYDWG-KFYASKTFYDHAKKRRILWSWIGETDSETAD-IAKGWASL
O81118          D-----FPDNDYGLRYDYG-DFYASKSFYDPVKKRRVLWGWANESDTVPDD-RNKGWAGI
O81985          PE---LDVGIG--LRCDYG-RFFASKSLYDPLKKRRITWGYVGESDSADQD-LSRGWATV
O81986          PE---NDVGIG--LRYDFG-KFYASKTFYDQHKKRRVLWGYVGETDPQKYD-LSKGWANI
O82119          T-----SIDGWKGLGLDYG-NYYASKTFFDSGKNRRILLGWANESDTVDND-VKKGWAGV
Q05G13          PE---ADLGIG--LRYDWG-KYYASKSFYDPIKNRRVVWAFVGETDSEQAD-KAKGWASL
Q05JI1          PE---EDVGIG--LKWDYG-RYYASKSFYDPYKKRRIVWGWINETDTESDD-LEKGWASV
Q05JI2          PE---IDVGIG--IRYDYG-LFYASKTFYDQNKKRRVLWGWIGESDSEIAD-VKKGWASL
Q0PCC5          PE---LDVGIG--LRVDYG-RFFASKSLYDPLNKRRVTWGYVGESDSPEQD-VNRGWATI
Q0PCC7          PE---LDVGIG--YRCDYG-RFFASKSLYDPLKKRRVTWGYVGESDSADQD-LSRGWATI
Q0PCC8          PE---LDVGIG--YRCDYG-RFFASKSLYDPLKKRRVTWGYIGESDSPDQD-LSRGWATI
Q0PCC9          PE---LDLGMG--WRCDYG-RFFASKTLYDPLKKRRVTWGYVAESDSGDQD-RSRGWSNI
Q0W9N0          PI---IDVGIG--LRYDYG-NFYASKSFYDQEKKRRVLWAWIKETDSEAAD-ICRGWASL
Q1KL65          PE---LDCGIG--LKLDYG-KYYASKTFYDPKKQRRVLWGWIGETDSESAD-LQKGWASV
Q2UXF7          VDVERNADDCRTWRRFDYG-HVYASKSFFDSSKNRRVLWAWANESDSQDND-IARGWSGV
Q2WEC6          PE---LDVGIG--LRCDYG-RFFASKSLYDPLKKRRITWAYVAESDSLDQD-LSRGWAHV
Q2XQ19          PD---ADVGIG--LRYDWG-KFYASKTFYDPAKRRRVLWGWVGETDSERAD-VAKGWASL
Q2XQ21          P-----AGDEHH-LRYDYG-NFYASKTFYDPAKRRRILWGWANESDTAVDD-VAKGWAGI
Q3L7K5          G-----SVDNDSGLRYDYG-KFYASKTFFDNAKNRRILWGWINESSSVEHD-IEKGWSGV
Q41215          PE---LDCGIG--LRLDYG-KYYASKTFYDPKKERRVLWGWIGETDSESAD-LQKGWASV
Q41604          VE---ADVGIG--WRYDYG-KFYASKTFFDWAKGRRVLFGFTGETDSEQNN-RLKGWASV
Q41606          VE---ADVGIG--WRYDYG-KFYASKTFFDWPKGRRVLFGFTGETDSEQNN-RLKGWASV
Q42691          G------FMNDSSLRYDYG-KYYASKTFYDGAKKERILLGWANESSSEEDD-AKKGWSGI
Q42722          PE---LDVGIG--LRYDYG-IYYASKTFYDSNKKRRVLWSWIKETDSEISD-VRKGWASV
Q43172          T-----SIDGWKGLRLDYG-NYYASKSFYDPSKNRRIMWGWANESDTVNDD-VKKGWAGI
Q43799          T-----SVDGWKGLRLDYG-NYYASKSFFDPSKNRRIMLGWANESDTVDDD-VRKGWAGV
Q43818          IL-----VDLVISERSLYG-KQVADKSILGLRKNDEALEMIASETPSVRNF-VSNGWLEI
Q43855          T-----SEDGWGGLRYDYG-NFYASKSFFDPTKNRRIIWGWANESDTKEDD-VKKGWAGI
Q43856          GF---EEVEIEEVFRYDYG-KYYASKTFFDYEKNRRILLGWVNESSIPDD-IKKGWSGI
Q4AEI9          TE---QELGVA--LRYDYG-RYDASKSFYDPVKQRRIVWGYVVETDSWSAD-AAKGWANL
Q547Q0          PE---LDCGIG--LRLDYG-KYYASKTFYDPKKERRVLWGWIGETDSESAD-LQKGWASV
Q575T1          SD---ADVGIG--LRYDWG-KFYASKTFYDPSKKRRVLWGWVGETDSEHAD-VAKGWASL
Q5ZQK6          -----GLKGNSLDMRYDYG-KFYASKSFYDNAKQRRVLWGWISEADAQEDD-VARGWSGL
Q64GB3          IVLD----DRRLLPRIDYG-NYYASKSFFDSKNRRRIIWGWTNESDSSSDD-VAKGWAGI
Q6F4N3          PE---ADLGIG--LRYDWG-KYYASKSFYDPIKNRRVVWAFVGETDSEQAD-KAKGWASL
Q6KCH6          VD---ADLGIG--LRYDWG-KFYASKTFYDPAKKRRVLWGWVGETDSESAD-VAKGWASL
Q6PVN1          AE---MDVGIG--LRYDWG-KFYASKTFYDPSKNRRVLWGWIGETDSERAD-VAKGWASL
Q70AT7          NVVD----DRRLWLRMDYG-TFYASKSFFDSKKGRRIVWGWSGETDSPSDD-LAKGWAGL
Q70LF5          DE---LELGVG--LRYDRG-KYYASKSFYDPVKKRRVVWACVGETDSERAD-ITKGWANL
Q70XE6          G-----SIESDLGLRYDYG-KFYASKSFFDDETNRRILWGWVNESSIQADD-IKKGWSGV
Q7DLY6          PS---IDVGISTGLRYDYG-KYYASKTFYDQNKGRRILWGWIGESDSEAAD-VQKGWSSV
Q7XA49          ------EESNEFVLRYDYG-KYYASKTFFDDGKKRRILLGWANESSSVAAD-IKKGWSGI
Q7XAS5          PE---LDCGIG--LKLDYG-KYYASKTFYDPKKQRRVLWGWIGETDSESAD-LQKGWASV
Q7XZS5          AE---KDVGTG--LRYDWG-KFYASKTFYDPAKRRRVLWGWVGKTDSERAD-FSKGWASL
```

```
Q84LA1          NVVD----DRRLWLRIDYG-TFYASKSFFDSNKNRRIIWGWSRETDSPSDD-LEKGWAGL
Q84RM0          RE---LDLGIG--LRYDWG-KYYASKSFYDQKKNRRIVWAYIGETDSEQAD-ITKGWANL
Q84V21          N-----PIDGWKGLRIDYG-NFYASKTFYDPSRNRRVIWGWSNESDVLPDDEIKKGWAGI
Q84XV1          T-----SADDHTGLRYDYG-NFYASKTFFDPKKQRRILWGWAKESDAEDVD-VAKGWAGI
Q8GT50          P-----AGDEHH-LRYDYG-NFYASKTFYDPVKRRRILWGWANESDAAVDD-VAKGWAGI
Q8GT63          DD-----EDCRSWRRLDYG-HVYASKSFFDPARTGACSGAGPTSPTACPTT-SSGDGPVF
Q8GUA3          PQ---LDVGIG--LRLDYG-KYYASKTFYDPKEQRRILWGWIGETDSEAAD-LLKGWASV
Q8GUB8          PE---NDLGIG--LRYDWG-KFYASKTFYDPAKQRRVLWGWIGETDSERAD-VAKGWASL
Q8L6W0          T----DFMEANTDWRYDYGGKFYASKTFFDGGKKRRILWAWIMEADSRAND-IKKGWSGL
Q8L6W1          G------YMNDSSLRYDYG-KYYASKTFFNDAKKERILLGWANESSSVEDD-IKKGWSGI
Q8L6W2          PA---IDVGIG--LRYDYG-RFYASKTFYDHEKKRRILWGWITEGDSEAAD-VKKGWASL
Q8L897          FK---NDVGVG--LRYDYG-IFYASKTFYDQKKNRRVLWGWIGESDSEYAD-VAKGWASV
Q8LPM7          DT---IDVGVG--LRYDYG-KFYASKTFYDQEKRRRILWGYVGEVDSKTDD-ARKGWANV
Q8LRN6          T-----SIDGWKGLRLDYG-NYYASKSFYDPSKNRRIVWGWANESDTVNDD-VKKGWAGI
Q8RVH4          PT---LDVGIG--LRYDYG-IYYASKTFYDQNKQRRVLWSWIKETDTEASD-IKKGWASL
Q8VXS5          G------YMNDSSLRYDYG-KYYASKPFFADAKKERILLGWANESSSVEDD-IKKGWSGI
Q8VXS7          PA---IDVGIG--LRYDYG-RFYASKTFYDHEKKRRILWGWITEGDSEAAD-VKKGWASL
Q8W3M2          PE---IDVGIG--IRYDYG-LFYASKTFYDQNKKRRVLWGWIGESDSEIAD-VKKGWASL
Q93X59          GL---SLTGSTLDLRYNYG-QFYASKSFFDDAKNRRVLWAWVPETDAPEDD-IEKGWAGL
Q941I4          PK---IDVGIG--LKYDYG-RYYASKTFYDQNKERRILLGWINETYTETDD-LEKGWSSL
Q944C8          PE---EDVGIG--LKWDYG-RYYASKSFYDPYKKRRIVWGWINETDTESDD-LEKGWASV
Q944U7          T-----LVDGWAGLRPDYG-NFYASKSFFDPKTNRRILWGWANESDSRQDD-VDKGWAGI
Q94C05          PE---IDVGIG--LRIDYG-KYYASKTFYDQNKGRRILWGWIGETDSEADD-LMKGWASV
Q94C06          TD---LDVGIG--LRLDYG-KYYASKTFYDQNKQRRILWGWIGETDLEAVD-LMKGWASL
Q94C07          AD---LDVGIG--LRLDYG-KCYAARTFYNQNKQRRILWGWIGETELEAVD-LMKGWASL
Q94C08          PD---LDVGIG--LRLDYG-KYYAARTFYNQNKQRRILWGWIGETDLEAVD-PMKGWASL
Q9AUH1          PE---LDLGIG--LRYDWG-KFYASTSFYDPAKKRRVLMGYVGEVDSKRAD-VVKGWASI
Q9FNS9          N----DFMVSNTQFQYDYG-RYYASKSFYDSVNQRRVIWGWVNEGDSESDA-VKKGWSGL
Q9FQ62          PT---IDVGMSASLRYDYG-KFYASKTFYDQNKGRRILWSWIGESDSEAAD-VQKGWSSL
Q9FR47          PE---LDLGMG--LRYDWG-KFYASTSFYDPIKRRRVVLGYVGETDSRRSD-EAKGWASI
Q9LD97          N-----SIDGWKGLRIDYG-NFYASKTFYDPSRNRRVIWGWSNESDVLPDDEIKKGWAGI
Q9LDS8          N-----SIDGWKGLRIDYG-NFYASKTFYDPSRNRRVIWGWSNESDVLPDDDIKKGWAGI
Q9M4K8          N-----SIDGSKGLRLDYG-NFYASKSFYDPMKNRRIVWGWTNESDVLPDDEIKKGWAGI
Q9SBI2          P-----AGDEHR-LRYDYG-NFYASKTFYDPAKRRRILWGWANESDTAADD-VAKGWAGI
Q9SM30          PE---NDVGIG--LRYDYG-KFYASKTFYDQHKSRRVLWGYVGETDPPKDD-LLKGWANM
Q9SPK0          P-----AGDEHR-LRYDYG-NFYASKTFYDPAKRRRILWGWANESDTAADD-VAKGWAGI
Q9ZP42          G-----SIESDSGLRYDYG-KFYASKTFFDSAKNRRILWGWINESSSVSGD-IKKGWSGL
Q9ZR55          EL---TLS----TLRLDYG-MYYASKSFFDPVKNRRIMTAWVNESDSEADV-IARGWSGV
Q9ZR96          PE---LDVGIG--LRVDYG-RFFASKSLYDPLKKRRVTWGYVAESDSADQD-LNRGWATI
Q9ZTW9          PD---LDVGIG--LRLDYG-KYYAARTFYDQNKQRRILWGWIGETDLEAVD-LMKGWASL
Q9ZTX2          TD---LDVGIG--LRLDYG-KYYASKTFYDQNKQRRILWGWIGETDLEAVD-LMKGWASL
P92916          ET---VDVGIG--LRYDWG-KFYASRTFFDPLKQRRIIWGYIGEVDSQKAD-IAKGWASL
Q5FC15          ES---VDVGIG--MRIDWG-KFYASRTFYDPVKERRVMWGYVGETDSGDAD-VAKGWASF
P93761          PD---LDCGIG--LRLDYG-RYYASKTFYDPKKQRRVLWGWIGETDSESAD-LQKGWASV
P49175          AE---DDVGIG--LRYDYG-KYYASKTFYDPVLRRRVLWGWVGETDSERAD-ILKGWASV
Q43089          G-----FEDNGFVLRYDYG-KYYASKTFFDDGKNRRILLGWVNESSSVADD-VKKGWSGI
Q39692          T-----SVDGWAGLRYDYG-NFYASKTFFDPDKQRRILWGWANESDSKQDD-VQKGWAGI
Q39693          T-----SVDGWAGLRYDYG-NFYASKTFYDPIKKRRILWGWANESDSQIDD-VQKGWAGI
Q56UD0          EDLEAHGDDYRRWRRIDHG-HLYASKTFYDAGKKRRVLWAWVNESDSEADD-VTKGWSGL
Q0J360          EP--ERGDDCRSWRRLDYG-HVYASKSFFDVRKNRRVLWAWANESDSQADD-VARGWSGV
P29001          VK---NDVGVG--LRYDYG-IFYASKTFYDQNKDRRILWGWIGESDSEYAD-VTKGWASV
O24509          VK---NDVGVG--LRYDYG-IFYASKTFYDQNMDRRILWGWIGESDSEYAD-VTKGWASV
P29000          PE---LDCGIG--LRLDYG-KYYASKTFYDPKKERRVLWGWIGETDSESAD-LQKGWASV
Q43857          FE---NDVGIG--LRYDYG-IFYASKTFYDQKKDRRVLWGWIGESDSEYAD-VAKGWASV
P80065          PE---LDVGIG--LRLDYG-KYYASKTFYDQDKERRLLWGWIGETDSESAD-LLKGWASV


Conservation:   0111102030111252749123
SS                  VC          VD
TARGET          QLIPRRVWLD-PSGKQLLQWPV
Q93X60          QSFPRALWID-RNGKQLIQWPV
Q43866          QTIPRKIWLD-RSGKQLIQWPV
A0A7Z0          QGIPRTVLFDKKTGSNLIQWPV
A3QRG0          MTIPRTVELDRKTRTNLIQWPV
A5GXL9          QSFPRSVWLS-DTRKQLVQWPV
A7IZK7          QAIPRLVWLD-KSGKQLVQWPI
A7IZK8          QTIPRTVVFDKKTGTNILQWPV
A7LJR5          EGLARTVLFDEKTGTNLLTWPV
A7RDD3          QNIPRTILFDTKTKSNLILWPV
A9CZQ1          QTVPRVLSVD-TDGKRLIQWPI
A9E2W4          QTVPRVLSLD-TDGKRLVQWPI
```

A9JIF3	QSFPRALWID-RSGMQLIQWPV
A9LST6	QSIPRTVLYDKKTGTHLLQWPV
A9YTS8	LNIPRSIVLDTQTGTNLIQWPI
A9YTS9	YNVARTVVLDRKTGTHLLHWPV
B0I1Q7	MSIPRTVELDKKTRTNLIQWPV
B2NIA0	HTIPRTVLFDSKTGTNLLQWPV
O04372	QGIPRTVLYDLRTGSNLITWPI
O23786	LNIPRTIVLDTKTNTNLIQWPI
O24459	LNIPRSVVLDTQTGTNLIQWPI
O65341	QGIPRTVLLDTKTGSNLLQWPV
O65342	QGIPRTVLLDTKTGSNLLQWPV
O65778	YNVARTIVLDRKTGTHLLQWPV
O81082	QNIARTILFDAKTRSNLLVWPV
O81083	QGVPRTVLLDVKTGSNLITWPV
O81118	QAIPRKIFLS-RSGRQLIQWPV
O81985	YNVGRTIVLDRKTGTHLLHWPV
O81986	LNIPRTVVLDLETKTNLIQWPI
O82119	HPIPRKIWLD-PSGKQLVQWPV
Q05G13	MSIPRTVELDKKTRTNLIQWPV
Q05JI1	QTIPRTVLYDNKTGSNVVQWPV
Q05JI2	QGIPRTVALDTKTGSNLLQWPV
Q0PCC5	YNVARTVVLDRKTGTHLLHWPV
Q0PCC7	YNVGRTVVLDRKTGTHLLHWPV
Q0PCC8	YNVARTVVLDRKTGTHLLHWPV
Q0PCC9	YNVARTVMLDRKTGTNLLQWPV
Q0W9N0	QPIPRTIQYDKKTGSNLITWPV
Q1KL65	QSIPRTVLYDKKTGTHLLQWPV
Q2UXF7	QTVPRKVWLD-EDGKQVRQWPI
Q2WEC6	YNVGRTIVLDRKTGTHLLHWPV
Q2XQ19	QSIPRTVVLDTKTGSNLLQWPV
Q2XQ21	QAIPRKVWLD-PSGKQLVQWPI
Q3L7K5	QAIPRNVWLD-KSGKQLLQWPI
Q41215	QSIPRTVLYDKKTGTHLLQWPV
Q41604	LPIPRTILFDQKTGSNLLLWPV
Q41606	LPIPRTILFDQKTGSNLLLWPV
Q42691	HTIPRTIWLD-KSGNQLIQWPI
Q42722	QGIPRTILFDPKTGSNLLQWPV
Q43172	QTIPRKLWLD-PSGKQLVQWPV
Q43799	HPIPRKLWLD-PSGKQLVQWPV
Q43818	Q-LDGNSLVTVERGKNRDGSVL
Q43855	QAIPRTVWLD-SSRRQLRQWPV
Q43856	HTIPRTIWLH-ESGKQLVQWPV
Q4AEI9	QSIPRTVELDEKTRTNLIQWPV
Q547Q0	QSIPRTVLYDKKTGTHLLQWPV
Q575T1	QSIPRTVVLDTKTGSNLLQWPV
Q5ZQK6	QAVPRSVWLD-RNGKQLVQWPV
Q64GB3	YGIPRTIWLD-RDGKQLLQWPV
Q6F4N3	MSIPRTVELDKKTRTNLIQWPV
Q6KCH6	QSTPRAVVLDTKTGSNLLQWPV
Q6PVN1	QSIPRTVELDEKTRTNLIQWPV
Q70AT7	HTIPRTIWLA-ADGKQLLQWPV
Q70LF5	QSIPRTVELDEKTRTNLIQWPV
Q70XE6	QAIPRTVVLD-KSGKQLVQWPL
Q7DLY6	QGIPRTVVLDTRTHKNLVQWPV
Q7XA49	HTIPRALWLH-KSGKQLVQWPV
Q7XAS5	QSIPRTVLYDKKTGTHLLQWPV
Q7XZS5	QGIPRTVLLDTKTGSNLLHWPV
Q84LA1	HTIPRTIWLA-GDGKQLLQWPV
Q84RM0	MTIPRTVELDRKTRTNLIQWPV
Q84V21	QGIPRQVRLN-LSGKQLLQWPI
Q84XV1	QAIPRTIWLD-SSGRQLIQWPI
Q8GT50	QAIPRKVWLD-PSGRQLMQWPV
Q8GT63	KLFPRKIWLD-EDGKQLRQWPV
Q8GUA3	QSIPRTVLYDKETRTHVLQWPV
Q8GUB8	MSIPRTVELDEKTRTNLIQWPV
Q8L6W0	QSIPRVVWLS-ASGNQLMQWPV
Q8L6W1	HTIPRKIWLD-KLGKQLIQWPI
Q8L6W2	QGIPRTVLFDQKTRTNLVQWPV
Q8L897	QSIPRIVKLDKKTGSNLLQWPV
Q8LPM7	LNIARTILFDVKTRSNLLVWPV
Q8LRN6	QTIPRKIWLD-SSGKQLVQWPV

Q8RVH4	MGVPRTIVLDKKTQSNIIQWPV
Q8VXS5	HTIPRKIWLD-KLGKQLIQWPI
Q8VXS7	QGIPRTVLFDQKTRTNLVQWPV
Q8W3M2	QGIPRTVALDTKTGSNLLQWPV
Q93X59	QSFPRALWID-RSGKQLIQWPI
Q941I4	QTIPRTVLFDNATGTQLLQWPV
Q944C8	Q---------------------
Q944U7	QTIPRKVWLD-PSGKQLRLWPV
Q94C05	QTIPRTVVLDTKTYTHLLQWPV
Q94C06	QAIPRTIVFDKKTGTNVLQWPV
Q94C07	QAIPRTMVFDEKTGTNVLQRPE
Q94C08	QAIPRSIVFDKKTGTNVLQRPE
Q9AUH1	QSVPRTIALDEKTRTNLLLWPV
Q9FNS9	QSFPRSIWLS-NNRKQLVQWPV
Q9FQ62	QGIPRTVVLDTKTGKNLVQWPV
Q9FR47	QSIPRTVALDKKTWTNLLLWPV
Q9LD97	QGIPRQVWLN-LSGKQLLQWPI
Q9LDS8	QGIPRQVWLD-LSGKQLVQWPI
Q9M4K8	QAIPRKVWLD-PSGKQLIQWPI
Q9SBI2	QAIPRTVWLD-PSGKQLLQWPI
Q9SM30	LNIPRTIVLDTVTGTNLIQWPI
Q9SPK0	QAIPRTVWLD-PSGKQLLQWPI
Q9ZP42	QAIPRTIVLD-KSRKQLVQWPV
Q9ZR55	QSFPRSLWLD-KNQKQLLQWPI
Q9ZR96	YNVARTIVLDRKTGTHLLHWPA
Q9ZTW9	QAIPRTIVFDKKTGTNVLQRPE
Q9ZTX2	QAIPRTIVFDKKTGTNVLQWPV
P92916	QGIPRSVLYDVKTGTNVLTWPI
Q5FC15	QGIPRTVLFDVKTGTNVLTWPI
P93761	QSIPRTVLFDKKTGTHLLQWPV
P49175	QSIPRTVLLDTKTGSNLLQWPV
Q43089	HTIPREIWLH-ESGKQLVQWPV
Q39692	QLIPRKLWLD-PNGKQLIQWPI
Q39693	QLIPRRIWLD-PSGRQLVQWPI
Q56UD0	QSFPRAVWLD-EGGRQLVQWPV
Q0J360	QTFPRKMWLA-KDGKQLLQWPI
P29001	QSIPRTVRLDTKTGSNLLQWPV
O24509	QSIPRTVRLDKKTGSNLLQWPV
P29000	QSIPRTVLYDKKTGTHLLQWPV
Q43857	QSIPRIVKLDKKTGSNLLQWPV
P80065	QSIPRTVVFDKKTGTNILQWPV
