# Supplementary material for: Integrating Bioinformatics Tools to Handle Glycosylation
Source: PLoS Comput Biol. 2011 Dec 29;7(12):e1002285. doi: 10.1371/journal.pcbi.1002285 (PMC3248387; doi:10.1371/journal.pcbi.1002285)
Supplement: Figure S3 — Pairwise sequence alignment. (PDF) [file pcbi.1002285.s003.pdf]

```
Template        MTKEVCSNIGLWL-LLTLLIGNYVVN---LEASHHVYKRLTQSTNTKSPSVNQPYRTGFH
Target          MDKLLGTALLKFLPVLPLFALLFVLSNNGVEASHKIYLRYQSLSVDK---VKQIHRTGYH
                * * : : :  :* :*.*:   :*:.   :****::* *  . :  *   *:* :***:*

Template        FQPPKNWMNDPNGPMIYKGIYHLFYQWNPKGAVWGNIVWAHSTSTDLINWDPHPPAIFPS
Target          FQPPKNWINDPNGPLYYKGLYHLFYQYNPKGAVWGNITWAHSVSKDLINWESLEPAIYPS
                *******:******: ***:******:**********.****.*.*****:.  ***:**

Template        APFDINGCWSGSATILPNGKPVILYTG-IDPKNQQVQNIAEPKNLSDPYLREWKKSPLNP
Target          KWFDNYGCWSGSATILPNGEPVIFYTGIVDGNNRQIQNYAVPANSSDPYLREWVKPDDNP
                  **  *************:***:*** :* :*:*:** * * * ******** *.  **

Template        LMAPDAVNGINASSFRDPTTAWLGQDKKWRVIIGSKIHRRGLAITYTSKDFLKWEKSPEP
Target          IVYPDPS--VNASAFRDPTTAWR-VGGHWRILIGSKKRDRGIAYLYRSLDFKKWFKAKHP
                :: **.   :***:********   . :**::**** : **:*  * * ** ** *: .*

Template        LHYDDGSGMWECPDFFPVTRFGSNGVETSSFGEPNEILKHVLKISLDDTKHDYYTIGTYD
Target          LHSVQGTGMWECPDFFPVSLSGEEGLDTS-VGGSN--VRHVLKVSLDLTRYEYYTIGTYD
                **  :*:***********:  *.:*::** .* .*  ::****:*** *:::********

Template        RVKDKFVPDNGFKMDGTAPRYDYGKYYASKTFFDSAKNRRILWGWTNESSSVEDDVEKGW
Target          EKKDRYYPDEALVDGWAGLRYDYGNFYASKTFFDPSKNRRILWGWANESDSVQQDMNKGW
                . **:: **:.:  . :. *****::********.:*********:***.**::*::***

Template        SGIQTIPRKIWLDRSGKQLIQWPVREVERLRTKQVKNLRNKVLKSGSRLEVYGVTAAQAD
Target          AGIQLIPRRVWLDPSGKQLLQWPVAELEKLRSHNVQ-LRNQKLYQGYHVEVKGITAAQAD
                :*** ***::*** *****:**** *:*:**:::*: ***: * .* ::** *:******

Template        VEVLFKVRDLEKADVIEPSWT--DPQLICSKMNVSVKSGLGPFGLMVLASKNLEEYTSVY
Target          VDVTFSFPSLDKAEPFDPKWAKLDALDVCAQKGSKAQGGLGPFGLLTLASEKLEEFTPVF
                *:* *.. .*:**: ::*.*:  *.  :*:: . ..:.*******:.***::***:*.*:

Template        FRIFKARQNSNKYVVLMCSDQSRSSLKEDNDKTTYGAFVDIN-PHQPLSLRALIDHSVVE
Target          FRVFKA---ADKHKVLLCSDARSSSLGEGLYKPPFAGFVDVDLTDKKLTLRSLIDHSVVE
                **:***   ::*: **:***   *** *.  *..:..***:: ..: *:**:********

Template        SFGGKGRACITSRVYPKLAIGKSSHLFAFNYGYQSVDVLNLNAWSMNSAQIS-----
Target          SFGAGGRTVITSRVYPIIAVFEKAHLFVFNNGSETVTVESLDAWSMKMPVMNVPVKS
                ***. **: ******* :*: :.:***.** * ::* * .*:****: . :.
```
