# Supplementary material for: Integrating Bioinformatics Tools to Handle Glycosylation
Source: PLoS Comput Biol. 2011 Dec 29;7(12):e1002285. doi: 10.1371/journal.pcbi.1002285 (PMC3248387; doi:10.1371/journal.pcbi.1002285)
Supplement: Text S1 — Supporting information text. (DOC) [file pcbi.1002285.s005.doc]

## Text S1.Insertion of *N*-glycan sites in invertase from *Populus alba* x *Populus grandidentata* using bioinformatics tools.

Bellow, an example to illustrate how to apply the workflow described in the manuscript for the rational design and insertion of *N*-glycan sites in proteins is provided. The cell wall invertase from *Populus alba x Populus grandidentata* was used as target for the introduction of *N*-glycosylation motifs. Cell wall invertase from *Populus alba x Populus grandidentata* (inv-Pa) belongs to the Glycosyl Hydrolase family 32 (GH32). GH32 comprises acid-type invertases (cell wall and vacuolar type in plants), fungal and bacterial endo and exo-inulinases, levanases, plant fructan exohydrolases, and plant fructan biosynthetic enzymes. Glycosyl hydrolase enzymes are important in cell wall metabolism, biosynthesis of glycans, plant defence, signalling, and mobilization of storage reserves. The overall three-dimensional (3D) structure of GH32 enzymes consists of an N-terminal fivefold β-propeller domain followed by a C-terminal domain named β-sandwich. Catalytic activity resides in the β-propeller domain. Such domain comprises five blades; each blade contains four antiparallel β-strands placed around a central axis [1]. Figure S1 shows the 3D structure of one of the members of GH32 protein family (invertase from *Arabidopsis thaliana*).

The aim is to introduce *N-*glycan sites in the inv-Pa catalytic domain (β-propeller). Note that, in this example we will go through the workflow knowing only the inv‑Pa amino acid sequence. In practice, one may already know the protein 3D structure, and may even have data from site-directed mutagenesis studies. Such information alerts for residues that should not be modified in order to preserve protein biological activity. The amino acid sequence of the inv-Pa was extracted from the UniProtKB database [2] (identification code B0LUL1):

>tr|B0LUL1| Cell-wall invertase OS=Populus alba x Populus grandidentata

MDKLLGTALLKFLPVLPLFALLFVLSNNGVEASHKIYLRYQSLSVDKVKQIHRTGYHFQPPKNWINDPNGPLYYKGLYHLFYQYNPKGAVWGNIVWAHSVSKDLINWESLEPAIYPSKWFDNYGCWSGSATILPNGEPVIFYTGIVDGNNRQIQNYAVPANSSDPYLREWVKPDDNPIVYPDPSVNASAFRDPTTAWRVGGHWRILIGSKKRDRGIAYLYRSLDFKKWFKAKHPLHSVQGTGMWECPDFFPVSLSGEEGLDTSVGGSNVRHVLKVSLDLTRYEYYTIGTYDEKKDRYYPDEALVDGWAGLRYDYGNFYASKTFFDPSKNRRILWGWANESDSVQQDMNKGWAGIQLIPRRVWLDPSGKQLLQWPVAELEKLRSHNVQLRNQKLYQGYHVEVKGITAAQADVDVTFSFPSLDKAEPFDPKWAKLDALDVCAQKGSKAQGGLGPFGLLTLASEKLEEFTPVFFRVFKAADKHKVLLCSDARSSSLGEGLYKPPFAGFVDVDLTDKKLTLRSLIDHSVVESFGAGGRTVITSRVYPIIAVFEKAHLFVFNNGSETVTVESLDAWSMKMPVMNVPVKS

By looking at the target amino acid sequence, it is unable to know where the *N*-glycan sites can be inserted without the disruption of protein tertiary structure and function. But, the analysis of *N*-glycosylation sequons localization in homologue or related proteins sharing a similar fold with the target could be the solution.

**Step-1: Multiple sequence alignment**

A sequence similarity search by doing a pairwise sequence alignment is the way to find homologue proteins. Since we know that the target protein belongs to the GH32 enzymes, a subset of proteins from this family was chosen to study *N*-glycosylation pattern. Protein sequences were extracted from the UniProtKB database. Percentage sequence identities between the target protein and selected proteins from GH32 family are found in Table S1. The inv-Pa target protein and the selected subset of GH32 enzymes were multiple aligned using CLUSTALW server [3] (Figure S1. Ribbon representation of the tertiary structure of invertase from Arabidopsis thaliana. The N-terminal domain belongs to the fivefold β-propeller. Each blade is shown in a different color: blade I (blue), blade II (red), blade III (yellow), blade IV (green) and blade V (pink). Strands are labelled A, B, C and D from the inside of the β-propeller outwards. The C-terminal β-sandwich domain is depicted in pum. The short polypeptide chain connecting the two domains is shown in dark gray. The picture was created using Chimera software [6].). In this point, the 3D structure availability of the homologues has to be checked, because it is used in further steps.

**Step-2: Sequence conservation analysis**

Next, the multiple sequence alignment is provided as input to perform sequence conservation analysis using the AL2CO server [4]. In the Figure S2, calculated conservation indices appear at the begging of each line in the multiple alignment with the heading “Conservation”. Conserved residues corresponding to the motifs: WMNDPNG, EC and RDP in the N*-*terminal domain (β-propeller domain) containing the catalytic triad were identified (Figure S2).

**Step-3: *N-*glycosylation sites prediction**

Now, we will search for *N*-glycan sites within protein sequences using the NetNGlyc server [5]. Possible occupied *N-*glycan sites (score > 0.5) were highlighted in red color in the multiple sequence alignment (Figure S2). *N*-glycosylation predictions suggested that in the catalytic domain of the GH32 protein family the major number of *N*-glycan sites resides in loops connecting β-strands. For example, around 70 *N*-glycosylation sequons are found in loops connecting β-strands C and D from Blade-II.

**Step-4: Insertion of *N*-glycan site**

After the analysis of the *N*-glycosylation pattern in GH32 protein family, an attractive position for the insertion of *N*-glycan sites in the target protein was identified. *N*-glycosylation site placed in the loop linking β-strands B and C from Blade-I is frequently observed among GH32 proteins (Figure S1. Ribbon representation of the tertiary structure of invertase from Arabidopsis thaliana. The N-terminal domain belongs to the fivefold β-propeller. Each blade is shown in a different color: blade I (blue), blade II (red), blade III (yellow), blade IV (green) and blade V (pink). Strands are labelled A, B, C and D from the inside of the β-propeller outwards. The C-terminal β-sandwich domain is depicted in pum. The short polypeptide chain connecting the two domains is shown in dark gray. The picture was created using Chimera software [6].). This *N*-glycan site has a high probability of been occupied by carbohydrates according to NetNGlyc server predictions. However, such *N*-glycan site is absent in the target protein. Then, the loop connecting β-strands B and C from Blade-I was selected to insert *N*-glycan site in the inv-Pa target protein.

In inv-Pa target protein, the insertion of the *N*-glycan site requires minor changes: (a) 93-NIV-95 (wild-target protein) changes to 93-NIS-95 or (b) 93-NIV-95 (wild-target protein) changes to 93-NIT-95. Replacement of Valine with Threonine residue is preferred. There is a high appearance frequency of Threonine residues compared to Serine in occupied *N*-glycan sites in position +2 [6]. Amino acid occupying position +1 in the new *N*-glycan site (93-NIT-95) is conserved among GH32 protein family, and then no changes are needed.

**Step-5: Modeling Target protein with inserted *N*-glycan site**

No 3D structure of the inv-Pa is available. Only 3D structures of cell-wall invertase 1 from *Arabidopsis thaliana* (Q43866) and fructan 1-exohydrolase IIa from *Cichorium intybus* (Q93X60) are resolved. Among proteins with available 3D structures, the cell-wall invertase 1 from *Arabidopsis thaliana* shares the highest percentage of sequence identity (54%). Then, a 3D structure model of the mutant inv-Pa (having new *N*-glycan sequon) using as template the cell-wall invertase 1 from *Arabidopsis thaliana* was built by homology modeling. The web-online SWISS-MODEL server [7] can be used, having as input the target-template alignment (Figure S3).

**Step-6: Addition of *N*-glycan molecules to the mutant target 3D model**

For the addition of the *N*-glycan molecules, the GlyProt server [8] was used, having the mutant 3D modeled structure of inv-Pa target protein as input. The new *N*-glycan site is exposed to the solvent (Figure S4). However, the *N*-glycan site is at the entrance of the active site, and it might block the cleft and interferes the substrate binding depending of the Asparagine conformations adopted. Then, the insertion of *N*-glycan site in other loops is recommended. For example, *N*-glycosylation sites observed in homologue proteins but away from the active site might be new attractive positions to explore.

**References**

1. Lammens W, Le Roy K, Schroeven L, Van Laere A, Rabijns A et al. (2009) Structural insights into glycoside hydrolase family 32 and 68 enzymes: functional implications. J Exp Bot 60: 727-740.

2. UniProt Consortium (2011) Ongoing and future developments at the Universal Protein Resource. Nucleic Acids Res 39: D214-D219.

3. Thompson JD, Higgins DG, Gibson TJ (1994) CLUSTAL W: improving the sensitivity of progressive multiple sequence alignment through sequence weighting, position-specific gap penalties and weight matrix choice. Nucleic Acids Res 22: 4673-4680.

4. Pei J, Grishin NV (2001) AL2CO: calculation of positional conservation in a protein sequence alignment. Bioinformatics 17: 700-712.

5. Gupta R, Jung E, Brunak S (2004) Prediction of N-glycosylation sites in human proteins.Available:http://www.cbs.dtu.dk/services/NetNGlyc/

6. Pettersen EF, Goddard TD, Huang CC, Couch GS, Greenblatt DM et al. (2004) UCSF Chimera--a visualization system for exploratory research and analysis. J Comput Chem 25: 1605-1612.

7. Schwede T, Kopp J, Guex N, Peitsch MC (2003) SWISS-MODEL: An automated protein homology-modeling server. Nucleic Acids Res 31: 3381-3385.

8. Bohne-Lang A, der Lieth CW (2005) GlyProt: in silico glycosylation of proteins. Nucleic Acids Res 33: W214-W219.

**Figure S1**. Ribbon representation of the tertiary structure of invertase from *Arabidopsis thaliana*. The N-terminal domain belongs to the fivefold β-propeller. Each blade is shown in a different color: blade I (blue), blade II (red), blade III (yellow), blade IV (green) and blade V (pink). Strands are labelled A, B, C and D from the inside of the β-propeller outwards. The C-terminal β-sandwich domain is depicted in pum. The short polypeptide chain connecting the two domains is shown in dark gray. The picture was created using Chimera software [6].

**Figure S2**. **Multiple sequence alignment of enzymes from GH32 protein family.** Only amino acid sequences from the catalytic domain are shown. Catalytic residues contained in the motifs: WMNDPNG, EC and RDP are denoted in yellow. Colors blue, red, yellow, green and pink denote each of the five blades. β-strands are labeled as A, B, C and D from the inside of the β-propeller outwards. For example, β-strand named ‘IIA’ corresponds to β-strand ‘A’ from Blade II. Secondary structure (in particular, β-strands) within the β-propeller domain is shown as rectangles at the second line of the alignment beginning with “SS”. Such data was extracted from available 3D structures of two GH32 proteins: cell wall invertase 1 from *Arabidopsis thaliana* (PDB code: 2AC1) and fructan 1-exohydrolase IIa from Cichorium intybus (PDB code: 1ST8) using DSSP software. Conservation indices for each aligned position are shown in the line beginning with “Conservation”. The attractive site for the insertion of *N*-glycan site is shadowed in cyan. Possible occupied *N*-glycan sites (score > 0.5) were highlighted in red color.

**Figure S3**. **Pairwise sequence alignment for homology modeling**. Pairwise sequence alignment between the cell wall invertase from *Populus alba* *x* *Populus grandidentata* (target) and cell wall invertase 1 from *Arabidopsis thaliana* (template).

**Figure S4.** Ribbon representation of the overall 3D structure of the mutant cell wall invertase model from *Populus alba x Populus grandidentata*. The N-terminal domain (β-propeller) is colored according to secondary structure features: β-strands in blue, helix in red and loops in light yellow. The C-terminal domain (β-sandwich) is shown in light green. β-strands B and C from Blade I, including the loop where the *N*-glycan site was inserted, are denoted in pink. The Asparagine residue side chain from the new *N-*glycosylation site (NIT) is colored in yellow. The attached *N-*glycan molecule is represented as sticks in orange color. Catalytic residues are shown in ball and sticks in black color. The picture was created using Chimera software [6].

**Table S1.** A subset of GH32 proteins and their corresponding score (or percentage of sequence identity) in relation with the inv-Pa target protein. The target protein is referred as ‘Target’ and the other proteins are named by their UniProtKB identification code. Proteins with resolved 3D structure are marked as ‘Yes’.

| Target | Homologue | Score | 3D |  | Target | Homologue | Score | 3D |
| --- | --- | --- | --- | --- | --- | --- | --- | --- |
| Target | Q39692 | 68 |  |  | Target | Q05JI2 | 42 |  |
| Target | Q43799 | 68 |  |  | Target | Q8W3M2 | 42 |  |
| Target | Q8LRN6 | 68 |  |  | Target | P49175 | 42 |  |
| Target | Q944U7 | 67 |  |  | Target | O04372 | 42 |  |
| Target | Q43855 | 67 |  |  | Target | Q9ZTX2 | 42 |  |
| Target | Q43172 | 66 |  |  | Target | Q9ZTW9 | 42 |  |
| Target | Q9M4K8 | 66 |  |  | Target | Q42722 | 42 |  |
| Target | Q39693 | 66 |  |  | Target | P93761 | 42 |  |
| Target | O82119 | 66 |  |  | Target | Q1KL65 | 42 |  |
| Target | Q9LDS8 | 65 |  |  | Target | Q7XAS5 | 42 |  |
| Target | Q9LD97 | 64 |  |  | Target | Q94C05 | 41 |  |
| Target | Q84V21 | 63 |  |  | Target | Q0W9N0 | 41 |  |
| Target | Q84XV1 | 59 |  |  | Target | Q8RVH4 | 41 |  |
| Target | Q8GT50 | 57 |  |  | Target | B2NIA0 | 41 |  |
| Target | Q7XA49 | 57 |  |  | Target | Q05JI1 | 41 |  |
| Target | Q9SBI2 | 56 |  |  | Target | Q41606 | 41 |  |
| Target | Q9SPK0 | 56 |  |  | Target | A7IZK8 | 41 |  |
| Target | Q2XQ21 | 56 |  |  | Target | O65342 | 41 |  |
| Target | O81118 | 56 |  |  | Target | O81083 | 40 |  |
| Target | Q9ZP42 | 55 |  |  | Target | Q941I4 | 40 |  |
| Target | Q3L7K5 | 55 |  |  | Target | Q9SM30 | 40 |  |
| Target | Q43866 | 54 | Yes |  | Target | Q41604 | 40 |  |
| Target | Q43856 | 54 |  |  | Target | Q0PCC5 | 40 |  |
| Target | A7IZK7 | 53 |  |  | Target | O81985 | 40 |  |
| Target | Q43089 | 53 |  |  | Target | O65341 | 40 |  |
| Target | Q8L6W1 | 52 |  |  | Target | Q8GUB8 | 40 |  |
| Target | Q8VXS5 | 51 |  |  | Target | Q94C07 | 39 |  |
| Target | Q70XE6 | 50 |  |  | Target | Q575T1 | 39 |  |
| Target | Q42691 | 50 |  |  | Target | Q94C08 | 39 |  |
| Target | Q9ZR55 | 50 |  |  | Target | Q6PVN1 | 39 |  |
| Target | Q64GB3 | 49 |  |  | Target | O24459 | 39 |  |
| Target | Q5ZQK6 | 48 |  |  | Target | Q7XZS5 | 39 |  |
| Target | Q9FNS9 | 48 |  |  | Target | O23786 | 39 |  |
| Target | Q0J360 | 47 |  |  | Target | O81986 | 39 |  |
| Target | Q8L6W0 | 47 |  |  | Target | A9YTS9 | 39 |  |
| Target | A9E2W4 | 47 |  |  | Target | Q0PCC8 | 39 |  |
| Target | A9CZQ1 | 47 |  |  | Target | Q944C8 | 39 |  |
| Target | A5GXL9 | 47 |  |  | Target | Q547Q0 | 39 |  |
| Target | Q70AT7 | 46 |  |  | Target | Q2XQ19 | 38 |  |
| Target | Q2UXF7 | 46 |  |  | Target | Q6KCH6 | 38 |  |
| Target | Q84LA1 | 46 |  |  | Target | A9YTS8 | 38 |  |
| Target | A9JIF3 | 46 |  |  | Target | Q8LPM7 | 38 |  |
| Target | Q93X60 | 46 | Yes |  | Target | O65778 | 38 |  |
| Target | Q93X59 | 46 |  |  | Target | Q2WEC6 | 38 |  |
| Target | Q43857 | 45 |  |  | Target | Q0PCC9 | 38 |  |
| Target | Q56UD0 | 45 |  |  | Target | O81082 | 37 |  |
| Target | A0A7Z0 | 44 |  |  | Target | Q9ZR96 | 37 |  |
| Target | P80065 | 44 |  |  | Target | P92916 | 37 |  |
| Target | Q94C06 | 44 |  |  | Target | Q5FC15 | 37 |  |
| Target | O24509 | 44 |  |  | Target | A3QRG0 | 36 |  |
| Target | P29001 | 44 |  |  | Target | Q84RM0 | 36 |  |
| Target | Q8L897 | 44 |  |  | Target | A7RDD3 | 36 |  |
| Target | Q7DLY6 | 44 |  |  | Target | B0I1Q7 | 35 |  |
| Target | A9LST6 | 44 |  |  | Target | A7LJR5 | 35 |  |
| Target | P29000 | 44 |  |  | Target | Q70LF5 | 35 |  |
| Target | Q8VXS7 | 43 |  |  | Target | Q9AUH1 | 35 |  |
| Target | Q8L6W2 | 43 |  |  | Target | Q4AEI9 | 34 |  |
| Target | Q9FQ62 | 43 |  |  | Target | Q05G13 | 34 |  |
| Target | Q8GUA3 | 43 |  |  | Target | Q6F4N3 | 34 |  |
| Target | Q41215 | 43 |  |  | Target | Q9FR47 | 34 |  |
| Target | Q8GT63 | 43 |  |  | Target | Q43818 | 20 |  |
| Target | Q0PCC7 | 43 |  |  |  |  |  |  |
